# Polymeric Photoacids Based on Naphthols—Design Criteria, Photostability, and Light‐Mediated Release

**DOI:** 10.1002/chem.201903819

**Published:** 2020-01-21

**Authors:** Felix Wendler, Maria Sittig, Jessica C. Tom, Benjamin Dietzek, Felix H. Schacher

**Affiliations:** ^1^ Institute of Organic Chemistry and Macromolecular Chemistry (IOMC) Friedrich Schiller University Jena Humboldtstrasse 10 07743 Jena Germany; ^2^ Jena Center for Soft Matter (JCSM) Friedrich Schiller University Jena Philosophenweg 7 07743 Jena Germany; ^3^ Institute of Physical Chemistry and Abbe Center of Photonics Friedrich-Schiller-University Jena Helmholtzweg 4 07743 Jena Germany; ^4^ Department of Functional Interfaces Leibniz Institute of Photonic Technology Jena Albert-Einstein-Strasse 9 07745 Jena Germany

**Keywords:** naphthol, photochemistry, photo-cleavage reactions, polymers, proton transfer

## Abstract

The implementation of photoswitches within polymers offers an exciting toolbox in the design of light‐responsive materials as irradiation can be controlled both spatially and temporally. Herein, we introduce a range of water‐soluble copolymers featuring naphthol‐based chromophores as photoacids in the side chain. With that, the resulting materials experience a drastic increase in acidity upon stimulation with UV light and we systematically studied how structure and distance of the photoacid from the copolymer backbone determines polymerizability, photo‐response, and photostability. Briefly, we used RAFT (reversible addition–fragmentation chain transfer) polymerization to prepare copolymers consisting of nona(ethylene glycol) methyl ether methacrylate (MEO_9_MA) as water‐soluble comonomer in combination with six different 1‐naphthol‐based (“N”) monomers. Thereby, we distinguish between methacrylates (NMA, NOeMA), methacrylamides (NMAm, NOeMAm), vinyl naphthol (VN), and post‐polymerization modification based on [(1‐hydroxynaphthalen‐2‐amido)ethyl]amine (NOeMAm, NAmeMAm). These P(MEO_9_MA_*x*_‐*co*‐“N”_*y*_) copolymers typically feature a 4:1 MEO_9_MA to “N” ratio and molar masses in the range of 10 kg mol^−1^. After synthesis and characterization by using NMR spectroscopy and size exclusion chromatography (SEC), we investigated how potential photo‐cleavage or photo‐degradation during irradiation depends on the type and distance of the linker to the copolymeric backbone and whether reversible excited state proton transfer (ESPT) occurs under these conditions. In our opinion, such materials will be strong assets as light‐mediated proton sources in nanostructured environments, for example, for the site‐specific creation of proton gradients. We therefore exemplarily incorporated NMA into an amphiphilic block copolymer and could demonstrate the light‐mediated release of Nile red from micelles formed in water as selective solvent.

## Graphical abstract

**Polymeric photoswitches**: A range of water‐soluble copolymers is reported featuring naphthol‐based chromophores as photoacids in the side chain. These materials experience a drastic increase in acidity upon stimulation with UV light and a systematic study was undertaken on how structure and distance of the photoacid from the copolymer backbone determines polymerizability, photo‐response, and photostability.

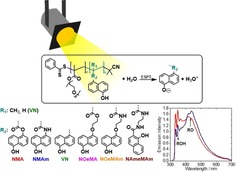

## Introduction

Various research fields benefit from advances in the design of molecular photoswitches,^1^ as light offers considerable advantages over other triggers (e.g., temperature or pH) including the possibility to control chemistry both spatially and temporally. Such photoswitches predominantly comprise organic chromophores such as azobenzenes,^2^ spiropyrans,^3^ and diarylethenes,^4^ and have so far been used in, for example, energy storage, chemical sensing, or in controlling both the conformation and the activity of biomolecules.^5^

Along the same lines, material and polymer science has been strongly affected by photoswitches,^6^ offering further possibilities to control macromolecular conformation, charge, or polarity with external triggers besides pH and temperature.^7^ Stimuli‐responsive materials are attractive for application in diagnostics, drug delivery, or tissue engineering and typical examples for light‐responsive polymers showed a shift in solubility, that is, in the hydrophobic‐hydrophilic balance upon irradiation.^8^ Early examples of photo‐responsive polymers featured azobenzenes in the side chain,^9^ or block copolymer micelles capable of undergoing reversible self‐assembly (disruption and reorganization) controlled via alternating UV and visible light exposure.^10^ Often, these materials are classified according to whether the underlying photochemical process is reversible or irreversible, in addition to synthetic access to different materials.^8^, ^11^ Especially the latter experienced a boost with the advent of controlled/“living” radical polymerization techniques such as atom transfer radical polymerization (ATRP),^12^ nitroxide‐mediated polymerization (NMP),^13^ and reversible addition–fragmentation chain transfer (RAFT) polymerization^14^—together with post‐polymerization modification of, for example, activated ester moieties if direct access to a certain photoswitch is hampered.^15^ Prominent examples of an irreversible photo‐response include photo‐cleavage of nitrobenzyl or pyrenyl esters and the formation of hydrophilic carboxylic acid groups along the polymer backbone,^16^ whereas a reversible photo‐response is often realized using diarylethenes, azobenzenes, or spiropyran moieties.^10^, ^17^

Besides changing local polarity or charge, it would nevertheless be beneficial if a photoswitch could additionally create a chemical gradient upon irradiation, as experienced in the case of proton gradients present in photosynthesis.^18^ In that respect, Meier and co‐workers recently demonstrated the successful insertion of the transmembrane protein proteorhodopsin as light‐activated proton pump into asymmetric polymersomes from an ABC triblock copolymer.^19^ Here, light absorption by this “proteopolymersome” induced pumping of protons into the vesicles, and resulted in an increase in the extravesicular pH, turning this into the first successful example of oriented insertion of a proton pump into an artificial asymmetric membrane.

An alternative for the creation of a local proton gradient are photoacids, which have been known since the seminal work of Förster^20^ and Weller^21^ in the 1960s. These photoswitches feature a protolytic group (hydroxyl group) that experiences a strong shift in acidity upon irradiation,^22^ and excited‐state proton transfer (ESPT) to the solvent can occur.^23^ In contrast to the main classes of photoswitches as discussed above, photoacids switch between ground and excited electronic states via irradiation with light of a specific wavelength, and each state establishes an equilibrium between the acid and its conjugate base. The rather generic term “photoacid” strictly applies to three different types of photoacids: excited‐state photoacids as described above, metastable‐state photoacids, and photoacid generators.^24^ Metastable‐state photoacids are usually based on spiropyran photochromes and are characterized by significantly higher lifetimes of the conjugate base if compared to excited‐state photoacids, rendering them suitable for applications in photo‐controlled proton‐sensitive processes.^25^ Photoacid generators typically exhibit irreversible photolysis upon irradiation releasing strong acids, and have been used mainly in lithographic processes.^26^ Although featuring rather short lifetimes of the corresponding conjugate bases, excited‐state photoacids have been investigated in the context of light‐triggered interactions with proteins,^27^ cyclodextrins,^28^ membranes,^29^, ^30^ and micelles.^30^, ^31^ The first known example of a polymeric excited‐state photoacid has been reported by Itoh et al., although the irradiation has not been studied.^32^ In 2017 we reported in a proof‐of‐concept study on the incorporation of 1‐naphthol‐based photoacids into statistical terpolymers and could show that irradiation with light led to a drastic increase in hydrophilicity.^33^

Herein, we introduce a set of well‐defined water‐soluble and light‐responsive polymeric photoacids, where the type and distance of the photoacid is systematically varied. Briefly, we synthesized copolymers consisting of nona(ethylene glycol) methyl ether methacrylate (MEO_9_MA) as a water‐soluble comonomer in combination with six different 1‐naphthol (“N”) derived monomers attached as either a methacrylate (NMA), a methacrylamide (NMAm), a methacrylate (NOeMA) or methacrylamide (NOeMAm) with an additional C_2_‐spacer, a vinylic (VN) group directly connected to the polymer backbone, and a monomer possessing amongst others, a divergent substitution pattern (NAmeMAm). These copolymers, namely P(MEO_9_MA_*x*_‐*co*‐“N”_*y*_) with a 4:1 MEO_9_MA to “N” ratio and molar masses in the range of 10 kg mol^−1^, were prepared using RAFT polymerization in combination with post‐polymerization modification of the activated ester copolymers. After synthesis and characterization by NMR spectroscopy and size exclusion chromatography, we carried out thorough investigations of the photochemical properties, with particular emphasis on the following scenarios:

1. Photo‐cleavage or photo‐degradation during irradiation in accordance with our previous assumptions and observations and depending on the type and distance of the linker to the polymeric backbone.^33^
2. Occurrence of the anticipated excited state proton transfer (ESPT) mechanism to provide reversibly light‐switchable and water‐soluble copolymers.

Besides providing guidelines on how to design polymerizable excited‐state photoacids and a more fundamental understanding of photo‐cleavage reactions during irradiation, we introduce a new type of stimuli‐responsive building block. With that, the herein derived materials can be used to introduce proton gradients within various types of nanostructured materials and open up applications to, for example, light‐mediated actuators or controlled release of encapsulated cargo. To demonstrate this, core‐corona micelles are formed in water from an amphiphilic block copolymer comprising both NMA and a sufficient amount of a protonizable comonomer in the hydrophobic segment. Upon irradiation, ESPT within the micellar core occurs, leading to a drastic increase in hydrophilicity, swelling, and release of Nile red as encapsulated model cargo.

## Results and Discussion

### Synthesis and characterization of photoacid‐containing water‐soluble copolymers

**Figure 1:**
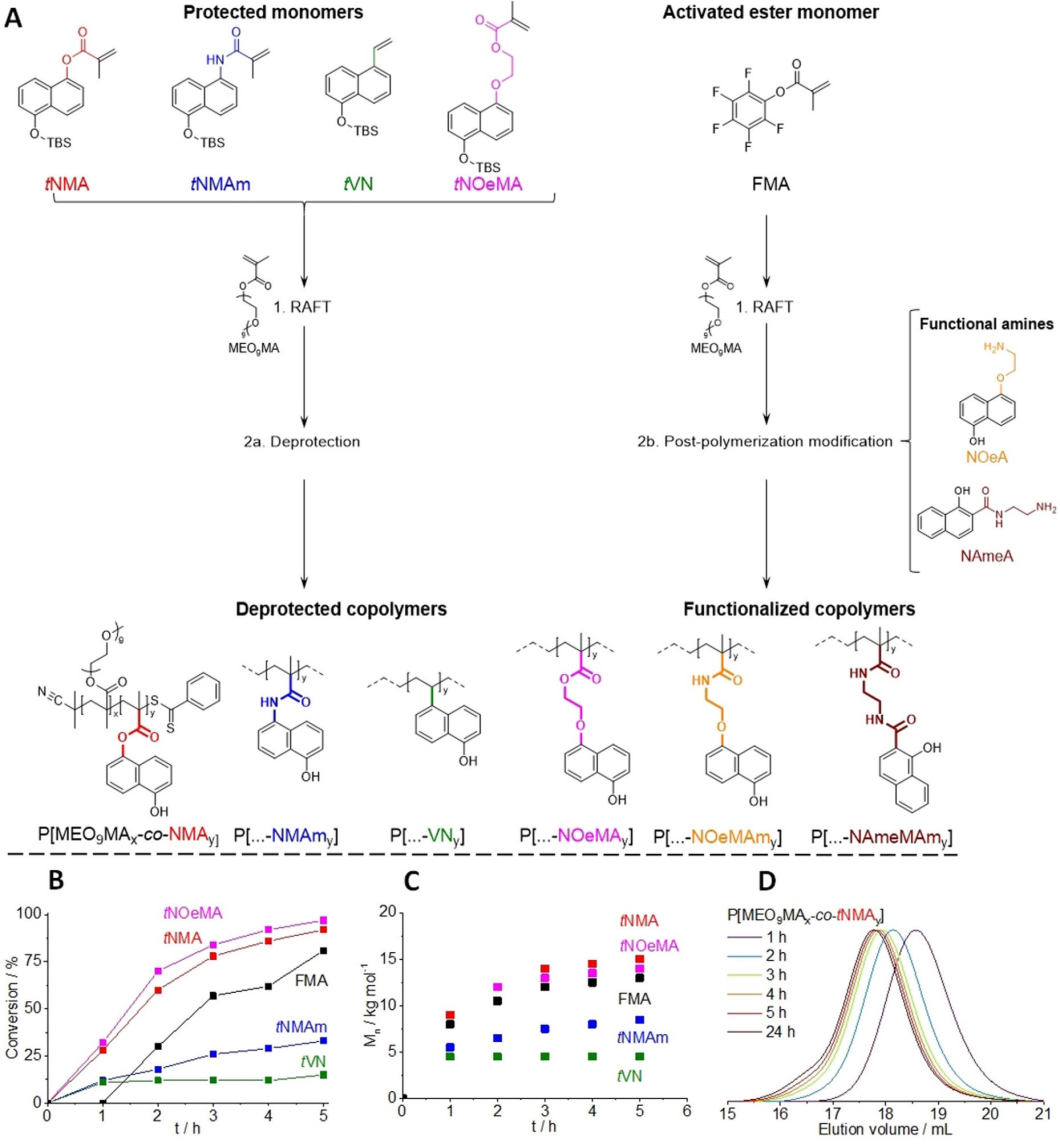
A) Synthetic pathway used to prepare six photoacid copolymers by either a direct RAFT polymerization (AIBN, CPDB, 1,4**‐**dioxane or bulk, 70 °C) of the respective functional comonomers followed by a deprotection step (2a), or through a post**‐**polymerization modification using an activated ester copolymer (P(MEO_9_MA**‐** *co* **‐**FMA)) and the respective functional amines (2b). B) Overall monomer conversion versus time plot for *t*NMA, *t*NMAm, *t*NOeMA, *t*VN, and FMA, copolymerized with MEO_9_MA. C) The corresponding *M_n_* versus time plot. D) SEC elution traces at different reaction times during the synthesis of P(MEO_9_MA_*x*_ **‐** *co* **‐** *t*NMA_*y*_).

Our aim was to prepare water‐soluble and well‐defined polymeric materials, which are capable of creating a local proton gradient upon irradiation with light of a suitable wavelength. We therefore designed six polymeric photoacids where the light‐responsive comonomer is in all cases based on 1‐naphthol,^34^ varying only in the type of attachment to and distance from the copolymer backbone. As the substitution pattern directly influences the electron density of the naphthol moiety, we expected this to also influence both the photo‐stability and photoacidity of the prepared materials. Furthermore, particular emphasis was put on the photoacidity under aqueous conditions, and therefore we chose nona(ethylene glycol) methyl ether methacrylate (MEO_9_MA) as the hydrophilic and water‐soluble comonomer. First, a set of copolymers containing 1‐naphthol derivatives substituted in position five were prepared leading to the respective methacrylate, that is, 1‐naphthol‐5‐methacrylate (NMA), and the respective methacrylamide (1‐naphthol‐5‐methacrylamide, NMAm, Figure 1 A). These two compounds have been the subject of a previous proof‐of‐concept study and we observed a certain amount of photocleavage or degradation during irradiation in deuterated DMSO.^33^ We extended this now to analogous compounds with an ethyl spacer in between the polymeric backbone and the 1‐naphthol (1‐naphthol‐5‐oxyethylmethacrylate (NOeMA) and 1‐naphthol‐5‐oxyethylmethacrylamide (NOeMAm)). Again, we are examining the difference between the presence of an ester and amide moiety (electrophilicity of the carbonyl carbon), as well as an aryl (‐I effect) versus alkyl (+I effect) substitution. Furthermore, we included NAmeMAm (1‐naphthol‐2‐oxyamidoethylmethacrylamide) possessing an *ortho*‐substitution of the naphthalene, incorporating a spacer between the chromophore and the polymeric backbone comprising an ethyl chain linked through an amide at each binding site. If we consider the naphthalene as a protecting group of the spacer, the *ortho*‐hydroxy group potentially leads to a significant increase in photo‐lability.^35^ Photochemical reactions of esters and amides are often described by Norrish‐type reactions involving α‐cleavage and we expect this to be more pronounced in the case of NAmeMAm due to the presence of two amide groups. Finally, we also included a vinyl‐based comonomer (5‐vinyl‐1‐naphthol, VN) where we anticipated a lower tendency for photo‐cleavage, as well as the suppression of undesirable photoreactions.

As shown in Figure 1 A, P(MEO_9_MA_*x*_‐*co*‐“N”_*y*_) copolymers in the case of NMA, NMAm, NOeMA and VN were prepared through RAFT copolymerization and subsequent deprotection of the respective functional comonomers. In order to prevent side reactions during the copolymerization process (e.g., inhibition, retardation), the hydroxyl groups were protected by using *tert*‐butyldimethylsilyl ether (TBS, herein abbreviated *t*), leading to the required deprotection step after polymerization. For NOeMAm and NAmeMAm, the respective P(MEO_9_MA_*x*_‐*co*‐“N”_*y*_) copolymers were not prepared through the polymerization of their respective methacrylamide monomers. Here, the corresponding amines, NOeA [2‐{(5‐hydroxynaphthalen‐1‐yl)oxy}ethyl]amine and NAmeA [{(1‐hydroxynaphthalen‐2‐amido)ethyl}amine] were used in a post‐polymerization modification involving the previously prepared activated ester copolymer P(MEO_9_MA‐*co*‐FMA).^36^ The synthesis of both the protected monomers and the functional amines is further outlined in the Supporting Information (Figure S1) including characterization by ^1^H NMR spectroscopy (Figure S2) and the synthetic procedures are described in more detail in the Experimental Section. The RAFT copolymerization of the protected monomers (*t*NMA, *t*NMAm, *t*NOeMA, and *t*VN) and activated ester monomer (FMA) was carried out using hydrophilic MEO_9_MA as mentioned previously. Briefly, AIBN was used as a thermal initiator at a reaction temperature of 70 °C with CPDB as an established chain transfer agent for the polymerization of methacrylates ([M]:[CTA]:[I]=25:1:0.25). For a better understanding regarding the incorporation of the respective comonomers within the copolymer, kinetic investigations for each monomer performed in 1,4‐dioxane as solvent were carried out.

Figure 1 B shows the overall monomer conversion over time of all five copolymerizations. For clarity, the respective individual conversions of both the functional comonomer and MEO_9_MA are omitted, but can be found in the Supporting Information (Figure S3). In general, no significant difference between the conversion of the functional comonomer and MEO_9_MA is observed (Figure S3). Except for *t*NMAm, MEO_9_MA is generally consumed more slowly than the functional comonomer as indicated by lower monomer conversions at any given time. Since an initial monomer feed ratio of MEO_9_MA to functional monomer of 4:1 is used in each case, we can presume that the copolymers formed are not strictly random. However, *t*NMA, *t*NOeMA, and FMA exhibit similar polymerization behavior as expected because they are all methacrylate‐based, and are almost completely consumed after 4 to 5 hours (81 to 97 % conversion), which is in close agreement with the half‐life of AIBN at 70 °C. In Figure 1 C, the molecular weight of each copolymer is observed to increase with reaction time, except in the case of *t*VN. In addition, the semi‐logarithmic plot (Figure S4) and the *M_n_* versus conversion plot (Figure S5) also exhibit linear correlations, which are good indicators for well‐controlled polymerization processes. This is further corroborated by narrow molecular weight distributions (*Ð*≤1.3), as highlighted in the SEC traces with increasing polymerization time (representatively shown for *t*NMA in Figure 1 D). However, after four hours of polymerization and conversions above 80 %, a higher molecular weight shoulder appears, which may indicate the occurrence of chain‐chain coupling. From these kinetic investigations, it can be stated that the copolymerizations of *t*NMA, *t*NOeMA, and FMA proceed in a controlled fashion, leading to molecular weights up to 15 kg mol^−1^ under these conditions.

As expected, the copolymerization behavior of *t*VN and *t*NMAm varied significantly. Figure 1 B reveals a very moderate overall monomer conversion, significantly lower than the values reached by the methacrylate‐based comonomers. *t*NMAm shows a linear increase in conversion (up to 35 %) and molecular weight (up to 9 kg mol^−1^) to a certain extent, as shown in Figure 1 C. However, the achieved overall monomer conversion of the *t*VN copolymerization is very low after one hour (ca. 10 %), and does not increase significantly, even after 24 h of copolymerization, reaching approximately 15 % conversion. This would correspond to approximately 8 repeating units based on the monomer feed ratio used, and the respective molar mass for such an oligomer is in the range of 3.5 kg mol^−1^. The molar masses obtained through SEC measurements at different reaction times are in agreement, with a molecular weight of approximately 4.5 kg mol^−1^ reached after one hour (Figure 1 C), and with no further significant increase observed by NMR after this time. Furthermore, moderate molecular weight distributions (*Ð*≤1.4) were obtained, and compared to the methacrylate comonomers, a high molecular weight shoulder is not observed. This is likely due to the significantly lower monomer conversions achieved and the outcome of both the semi‐logarithmic plot and *M_n_* versus conversion plot for *t*NMAm and *t*VN are shown in Figure S4 and Figure S5. Hence, the conditions reported herein are unsuitable to obtain higher molecular weight copolymers featuring *t*NMAm and *t*VN, and further optimization is reasonable, for example, by changing the utilized RAFT agent. Particularly in the case of *t*VN, it might be advantageous to switch to a styrenic‐based copolymer backbone in the future. Nevertheless, we have access to a set of well‐defined random copolymers with comparable molecular weights and photoacid content, with no indications thus far for a gradient structure.

Based on the above findings, we synthesized the final copolymers using similar reaction conditions (Table 1). The copolymerizations of *t*NMA, *t*NOeMA and FMA were terminated after three hours in order to prevent chain coupling and, hence, the monomer feed ratios were corrected according to the efficiency of the polymerization based on our kinetic investigations to target a molecular weight of approximately 10 kg mol^−1^. For *t*VN and *t*NMAm, no high molecular weight shoulders were observed, therefore it was not necessary to adjust the polymerization conditions. To improve the monomer conversions obtained and achieve higher molecular weight copolymers, copolymerizations were carried out for 24 h. For the same reason, bulk conditions were applied, and also the monomer feed ratio with respect to the RAFT agent was doubled to achieve higher degrees of polymerization. Detailed characterization data of the protected copolymers P[MEO_9_MA_*x*_‐*co*‐*t*“N”_*y*_] and P(MEO_9_MA_*x*_‐*co*‐PFMA_*y*_) is shown in Table 1. The five copolymers prepared had similar molar masses (ca. 9 kg mol^−1^), and the copolymer compositions obtained are in good agreement with the monomer feed ratios used. For P[MEO_9_MA_*x*_‐*co*‐PFMA_*y*_], it is more challenging to determine the copolymer composition as the PFMA repetition units cannot be visualized by ^1^H NMR besides the backbone signals. Despite this, the degree of polymerization of MEO_9_MA was calculated to be 29. This appears relatively high if compared to the adjusted value of 20 (according to the used monomer feed ratio) and the achieved molar mass from SEC measurements being in the expected range (10.5 kg mol^−1^).

**Table 1:** SEC and ^1^H NMR characterization of the protected copolymers, P[MEO_9_MA_*x*_‐*co*‐*t*“N”_*y*_] and P(MEO_9_MA_*x*_‐*co*‐FMA_*y*_), and for the respective deprotected or functionalized copolymers, P[MEO_9_MA_*x*_‐*co*‐“N”_*y*_].

Sample	*M*_n_^[a]^ [kg mol^−1^]	*Ð* ^[a]^	MEO_9_MA^[b]^ [%]	*t*“N”/FMA^[b]^ [%]
P[MEO_9_MA_*x*_‐*co*‐*t*NMA_*y*_]	9	1.18	80	20
P[MEO_9_MA_*x*_‐*co*‐*t*NMAm_*y*_]	8	1.15	77	23
P[MEO_9_MA_*x*_‐*co*‐*t*VN_*y*_]	7	1.20	75	25
P[MEO_9_MA_*x*_‐*co*‐*t*NOeMA_*y*_]	8	1.14	79	21
P[MEO_9_MA_*x*_‐*co*‐PFMA_*y*_]	10.5	1.30	–^[c]^	–^[c]^
P[MEO_9_MA_*x*_‐*co*‐NMA_*y*_]	13	1.13	81	19
P[MEO_9_MA_*x*_‐*co*‐NMAm_*y*_]	11.5	1.15	67	33
P[MEO_9_MA_*x*_‐*co*‐VN_*y*_]	10	1.14	78	22
P[MEO_9_MA_*x*_‐*co*‐NOeMA_*y*_]	11.5	1.13	78	22
P[MEO_9_MA_*x*_‐*co*‐NOeMAm_*y*_]	18	1.25	72	28
P[MEO_9_MA_*x*_‐*co*‐NAmeMAm_*y*_]	17.5	1.22	65	35

[a] SEC (DMAc/LiCl) (PMMA calibration). [b] ^1^H NMR (300 MHz, CD_2_Cl_2_). [c] The copolymer composition could not be determined by NMR in this case. The DP of MEO_9_MA is ≈29, and the MW determined by SEC is within the desired range for this study.

**Figure 2:**
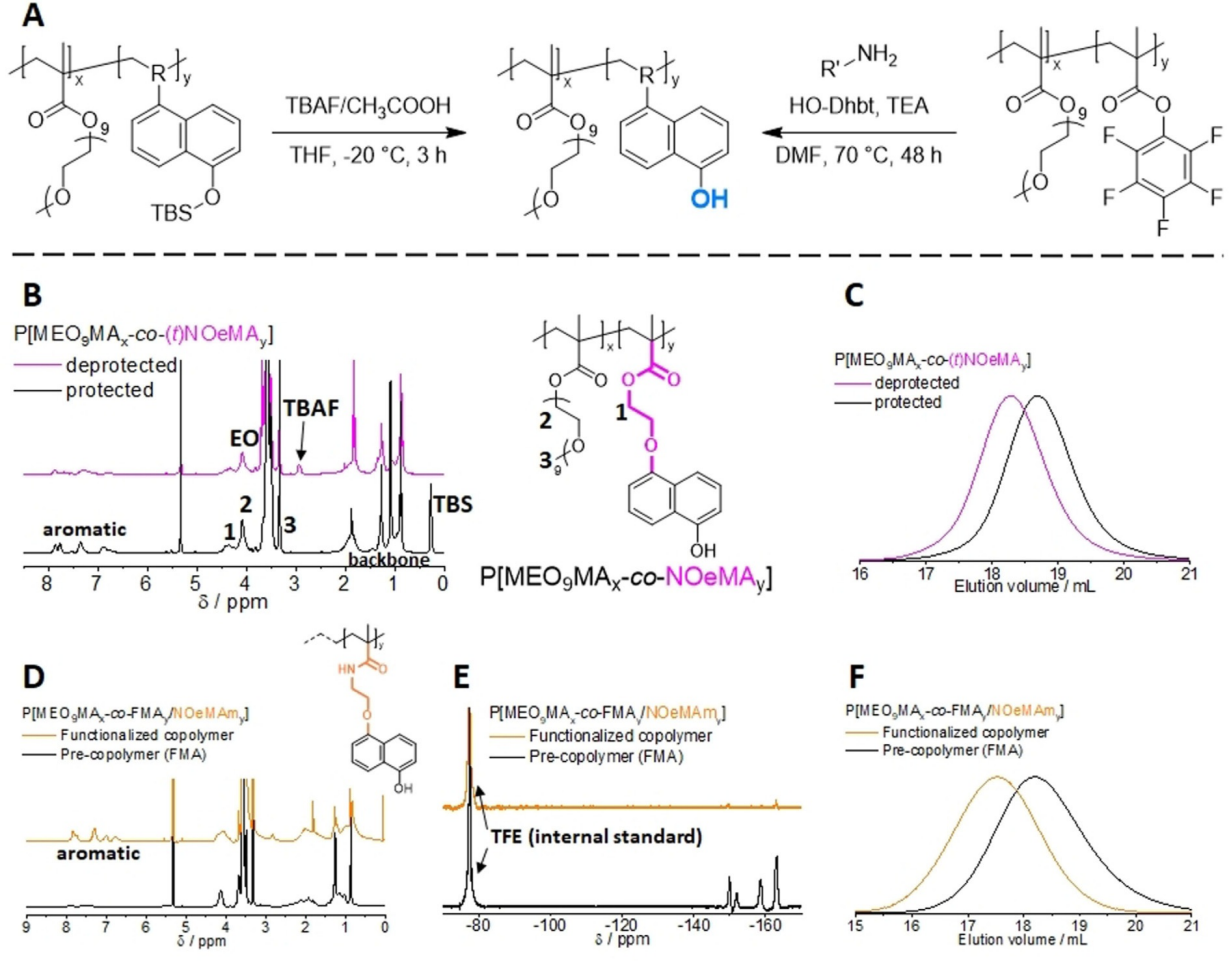
A) Synthesis of the photoacid**‐**containing copolymers through a direct deprotection of TBS protected copolymers, P[MEO_9_MA_*x*_ **‐** *co* **‐** *t*“N”_*y*_], or through a post**‐**polymerization modification of the activated ester copolymer P(MEO_9_MA_*x*_ **‐** *co* **‐**FMA_*y*_) and the respective photoacid functionalized amines (for clarity, the RAFT end groups of the copolymers were omitted here). B) Representative ^1^H NMR spectra of P[MEO_9_MA_*x*_ **‐** *co* **‐** *t*NOeMA_*y*_] before (solid black line) and after deprotection (solid magenta line) in CD_2_Cl_2_. C) Representative SEC elution traces for P[MEO_9_MA_*x*_ **‐** *co* **‐** *t*NOeMA_*y*_] before (solid black line) and after deprotection (solid magenta line). D) Representative ^1^H NMR spectra of P[MEO_9_MA_*x*_ **‐** *co* **‐**FMA_*y*_/NOeMAm_*y*_] before (solid black line) and after post**‐**polymerization modification (solid orange line) in CD_2_Cl_2_. E) Representative ^19^F NMR spectra of P[MEO_9_MA_*x*_ **‐** *co* **‐**FMA_*y*_/NOeMAm_*y*_] before (solid black line) and after post**‐**polymerization modification (solid orange line) in CD_2_Cl_2_ using TFE as an internal standard. F) Representative SEC elution traces for P[MEO_9_MA_*x*_ **‐** *co* **‐**FMA_*y*_/NOeMAm_*y*_] before (solid black line) and after post**‐**polymerization modification (solid orange line).

To obtain the final materials, the as‐synthesized P[MEO_9_MA_*x*_‐*co*‐*t*“N”_*y*_] were subjected to a deprotection step, whereas in the case of P[MEO_9_MA_*x*_‐*co*‐PFMA_*y*_], a post‐polymerization modification was carried out using the designed photoacid functionalized amines NOeA or NAmeA (Figure 2 A). The deprotection was carried out using a two‐fold excess of TBAF with respect to the *t*“N” repetition units in an equimolar combination with acetic acid according to a literature procedure.^37^

^1^H NMR spectroscopy was used to characterize both the protected and deprotected copolymers (Figure 2 B). The disappearance of the signal at 0.3 ppm corresponding to the protecting group indicates the successful removal of TBS. The SEC traces (Figure 2 C) show a clear shift towards higher molar masses as expected based on our previous observations; stronger hydrogen bonding and potential interactions with the SEC column due to the increased number of hydroxyl groups explain this behavior.^33^ In order to remove excess TBAF, preparative size exclusion chromatography (BioBeads™ SX‐1) with THF as eluent was performed. However, residual TBAF could be detected even after this purification process on several occasions. Other purification methods including precipitation and dialysis were also investigated, but did not always improve the purity. The final copolymer compositions were determined after both deprotection and purification and were in good agreement with the data obtained for the protected copolymers.

The post‐polymerization modification was carried out using a fivefold excess of the functional amines NOeA or NAmeA with respect to the incorporated FMA units, and an equimolar amount of trimethylamine and HO‐Dhbt as coupling reagent according to a literature procedure (Figure 2 A).^38^ The reaction was conducted in DMF at 70 °C for two days to ensure full conversion, and ^1^H NMR spectroscopy was used to characterize the final functionalized copolymers (Figure 2 D). The appearance of aromatic signals corresponding to the 1‐naphthol protons indicated a successful functionalization and the actual composition of both P[MEO_9_MA_*x*_‐*co*‐NOeMAm_*y*_] and P[MEO_9_MA_*x*_‐*co*‐NAmeMAm_*y*_] could be calculated and were 28 and 35 %, respectively. To further evaluate the extent of functionalization, ^19^F NMR spectroscopy was carried out and a representative ^19^F spectra for P[MEO_9_MA_*x*_‐*co*‐NOeMAm_*y*_] is shown in Figure 2 E (the respective ^19^F NMR spectra of P[MEO_9_MA_*x*_‐*co*‐NAmeMAm_*y*_] can be found in the Supporting Information, Figure S6). The characteristic fluorine signals expected for P[MEO_9_MA_*x*_‐*co*‐FMA_*y*_] are clearly visible before and absent after the reaction. Only very weak signals in the respective area of the spectrum remain, which correspond to a rather high degree of functionalization (>99 %). Finally, a significant shift is observable by SEC, which clearly indicates the expected increase in molecular weight as a result of this post‐polymerization modification (Figure 2 F).

### Photophysical properties of the 1‐naphthol‐based (“N”) photoacid comonomers and the corresponding copolymers

**Figure 3:**
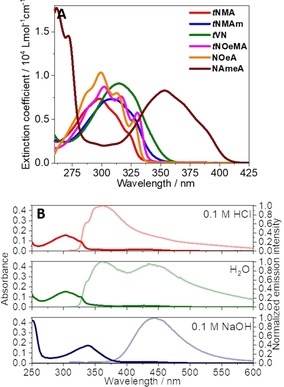
Absorption spectra of the protected photoacid comonomers (*t*“N”) and the functional amines in DMSO (A). UV/Vis absorption and emission (light colored) spectra of P[MEO_9_MA_*x*_ **‐** *co* **‐**VN_*y*_] in aqueous media: 0.1 m HCl (red), H_2_O (green) and 0.1 m NaOH (blue, B).

Due to the lack of solubility in H_2_O, the UV/Vis absorption properties of the protected photoacid comonomers (*t*“N”) and the functional amines were investigated in DMSO (Figure 3 A). While most 1‐naphthol derivatives absorb in the range below 350 nm, NAmeA shows a strong absorption at longer wavelengths ranging up to 420 nm. The main absorption band located around 300 nm (in the case of NAmeA around 350 nm) of the 1‐naphthol derived monomers exhibits molar extinction in the range of 7300 (*t*NMAm) to 10 400 (*t*NOeMA) m ^−1^ cm^−1^. The absorption of 1‐naphthol derivatives in this range is assigned to superimposed transitions in the first excited singlet state, ^1^L_b_, and to the second excited singlet state, ^1^L_a_.^34^

In case of the respective photoacid copolymers in aqueous solution, the protonated species, ROH, of the photoacid exhibits a absorption maxima in the range between 299 and 309 nm. P[MEO_9_MA_*x*_‐*co*‐NAmeMAm_*y*_] reveals a bathochromically shifted absorption peak at 342 nm and the conjugate base of the photoacid copolymers, RO^−^, generally exhibits a red‐shifted absorption. For the 1‐naphthol derivatives, the respective maxima are localized at approximately 339 nm (354 nm for P[MEO_9_MA_*x*_‐*co*‐NAmeMAm_*y*_]). The protonated form of the photoacid and the conjugate base show an emission centered at approximately 350 and 440 nm, respectively.

Figure 3 B exemplarily shows the absorption and fluorescence spectra of P[MEO_9_MA_*x*_‐*co*‐VN_*y*_] in aqueous environments at different pH values, that is, in 0.1 m HCl (red, fully protonated), H_2_O (green, pH 5.7) and 0.1 m NaOH (blue, fully deprotonated form). The dual emission observed for P[MEO_9_MA_*x*_‐*co*‐VN_*y*_] in water exhibits contributions from both the neutral, protonated form at 360 nm, and its conjugated, deprotonated form at 443 nm, clearly indicating the occurrence of an ESPT with water acting as a base. Most remaining photoacid copolymers qualitatively show similar behavior, which is summarized in Table 2 (the remaining steady‐state absorption and emission spectra are compiled in the Supporting Information; see Figures S7 and S8). An exception is P[MEO_9_MA_*x*_‐*co*‐NAmeMAm_*y*_], where no distinct change in emission upon pH changes was observed, hinting towards no photoacidity for the NAmeMAm chromophore upon optical excitation.

**Table 2:** Steady**‐**state spectral properties of the photoacid**‐**containing copolymers together with their estimated ground and excited state acidity constants under aqueous conditions, p*K*_a_ and p*K*_a_*, respectively.

Sample	$\lambda_{abs}^{max}$ [nm]		$\lambda_{em}^{max}$ [nm]		p*K*_a_^[a]^	p*K*_a_*^[b]^	p*K*_a_*^[c]^
	ROH	RO^−^	ROH	RO^−^			
P[MEO_9_MA_*x*_‐*co*‐NMA_*y*_]	302, 313, 326	339	348, 362	456	8.7	0.3	n.d.^[d]^
P[MEO_9_MA_*x*_‐*co*‐NMAm_*y*_]	309, 328	339	368	456	9.3	0.5	1.1
P[MEO_9_MA_*x*_‐*co*‐VN_*y*_]	303, 314, 327	338	360	443	9.4	0.8	1.8
P[MEO_9_MA_*x*_‐*co*‐NOeMA_*y*_]	299, 315, 329	339	333, 347, 363	433	9.7	2.9	2.4
P[MEO_9_MA_*x*_‐*co*‐NOeMAm_*y*_]	300, 315, 329	339	334, 347, 363	435	9.3	2.5	2.1
P[MEO_9_MA_*x*_‐*co*‐NAmeMAm_*y*_]	342, 352	354	431	418	8.9	–	–

[a] Estimated by using absorption titration. [b] Estimated by using the Förster cycle. [c] Evaluated by using emission titration. [d] n.d.=not determined, not accessible due to the formation of an unknown emissive photoproduct ($\lambda_{em}^{max}$ =515 nm).

### Determination of the ground and excited state acidity constants

**Figure 4:**
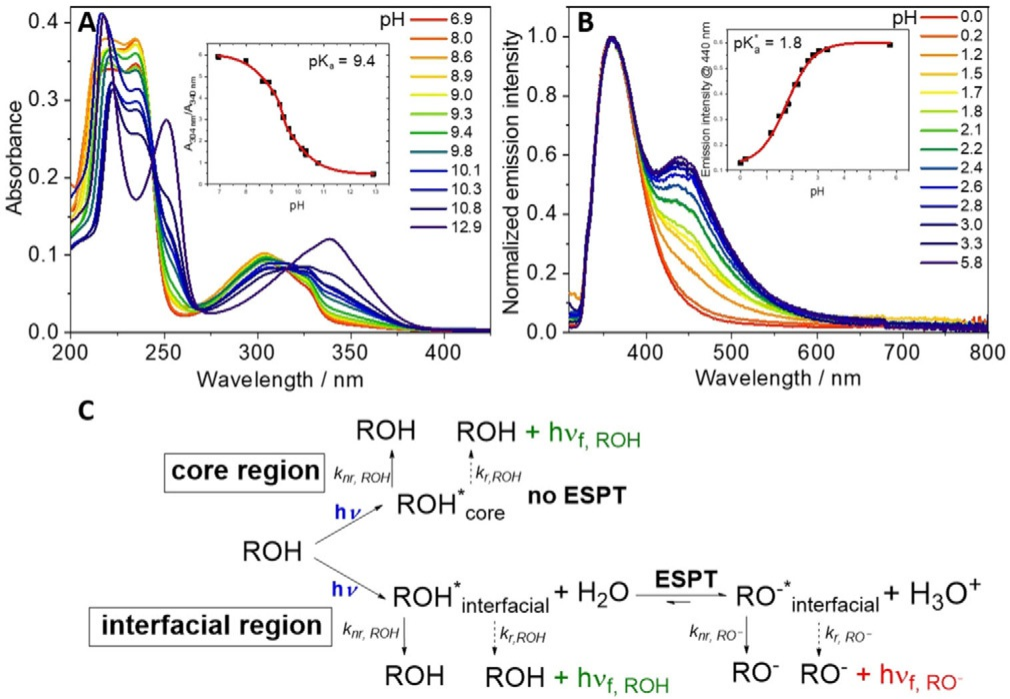
A) pH**‐**dependent UV/Vis absorption spectra and B) pH**‐**dependent normalized emission spectra of P[MEO_9_MA_*x*_ **‐** *co* **‐**VN_*y*_] in aqueous solution. The insets show the estimated p*K*_a_ and p*K*_a_* values. C) Possible photophysical processes upon excitation of 1**‐**naphthol in the photoacid copolymers within the core and interfacial regions. In this context *k*_nr_ and *k*_r_ refer to the respective non‐radiative and radiative decay rate constants of electronically excited protonated species ROH^*^ and deprotonated species RO^−*^.

The ground state acidity constant, p*K*_a_, of 1‐naphthol in the copolymers was determined by spectrophotometric titration and the values obtained range from 8.7 for P[MEO_9_MA_*x*_‐*co*‐NMA_*y*_] to 9.7 for P[MEO_9_MA_*x*_‐*co*‐NOeMA_*y*_] (see Figure 4 A and Figure S9), consistent with literature‐reported values.^39^

The change in acidity of the polymeric photoacids upon photoexcitation is determined using the Förster cycle analysis.^40^ Thereby, the change in p*K*_a_* units upon optical excitation is derived from the steady‐state absorption and fluorescence spectra by use of Equation (1):

(1) $$pK_{a}^{{}^{*}}=pK_{a}-\frac{N_{A}hc}{RTln10}\left({\tilde{\nu }}_{0-0\mathrm{ROH}}-{\tilde{\nu }}_{0-0RO^{-}}\right)$$

In this equation $N_{A}$ , h , c , R and T correspond to Avogadro's constant, Planck's constant, the speed of light, the universal gas constant, and the temperature. The wavenumber position of the 0‐0 electronic transition of the acid and base form, ${\tilde{\nu }}_{0-0\;\mathrm{ROH}}\;$ and $\;{\tilde{\nu }}_{{0-0\;\mathrm{RO}}^{-}}$ , are estimated from the intersection of the absorption and normalized fluorescence spectra of the corresponding acid and conjugate base forms.^41^ The p*K*_a_* of NMA, NMAm and VN were estimated to be 0.3, 0.5 and 0.8, respectively; whereas, NOeMA and NOeMAm show a slightly higher acidity constant with a p*K*_a_* of 2.9 and 2.5, respectively. The higher p*K*_a_* of the derivatives NOeMA and NOeMAm can be rationalized by considering the electron‐donating effect of the ether functionality used as a linker between the photoactive unit to the copolymer backbone. The change in p*K*_a_ units upon photoexcitation amounts to up to −8.6 p*K*_a_ units in P[MEO_9_MA_*x*_‐*co*‐NMA_*y*_], P[MEO_9_MA_*x*_‐*co*‐NMAm_*y*_] and P[MEO_9_MA_*x*_‐*co*‐VN_*y*_]. Despite the distinct increase in the acidity constant in the excited state, p*K*_a_*, full deprotonation of the “N” moieties in the copolymers is not observed under our experimental conditions. In our opinion, this can be explained with the ESPT being strongly dependent on the accessibility of a photoacid to water molecules.^42^, ^43^ One tentative explanation is that parts of the copolymers are not completely hydrated, creating regions where individual photoacid monomers are not accessible to water molecules, and we call these “core” regions. In this scenario, ESPT would occur predominantly in the interfacial region between the solvated copolymer and the surrounding medium (Figure 4 C).^43^

Fluorescence titration, on the other hand, allows for the direct estimation of the p*K*_a_* value, but bears certain challenges in environments where the deprotonation of the 1‐naphthol units is not fully observed. Here, we estimated the p*K*_a_* by normalizing the fluorescence spectra to the emission maxima of the protonated ROH form (see Figures 4 B and S9). Subsequently, the p*K*_a_* is derived from the increase in the RO^−^ emission located at approximately 450 nm as an inflection point of the sigmoidal curve and the values are in quite good agreement with the ones obtained by the Förster equation. Here, we found 1.1 and 1.8 for NMAm and VN in P[MEO_9_MA_*x*_‐*co*‐NMAm_*y*_] and P[MEO_9_MA_*x*_‐*co*‐VN_*y*_], whereas NOeMA and NOeMAm display a slightly lowered acidity with a p*K*_a_* of 2.4 and 2.1, respectively. The excited state acidity constant of NMA in P[MEO_9_MA_*x*_‐*co*‐NMA_*y*_] could not be determined due to the presence of an unknown emissive photoproduct ($\lambda_{\max}^{\mathrm{em}}$ =515 nm).

**Figure 5:**
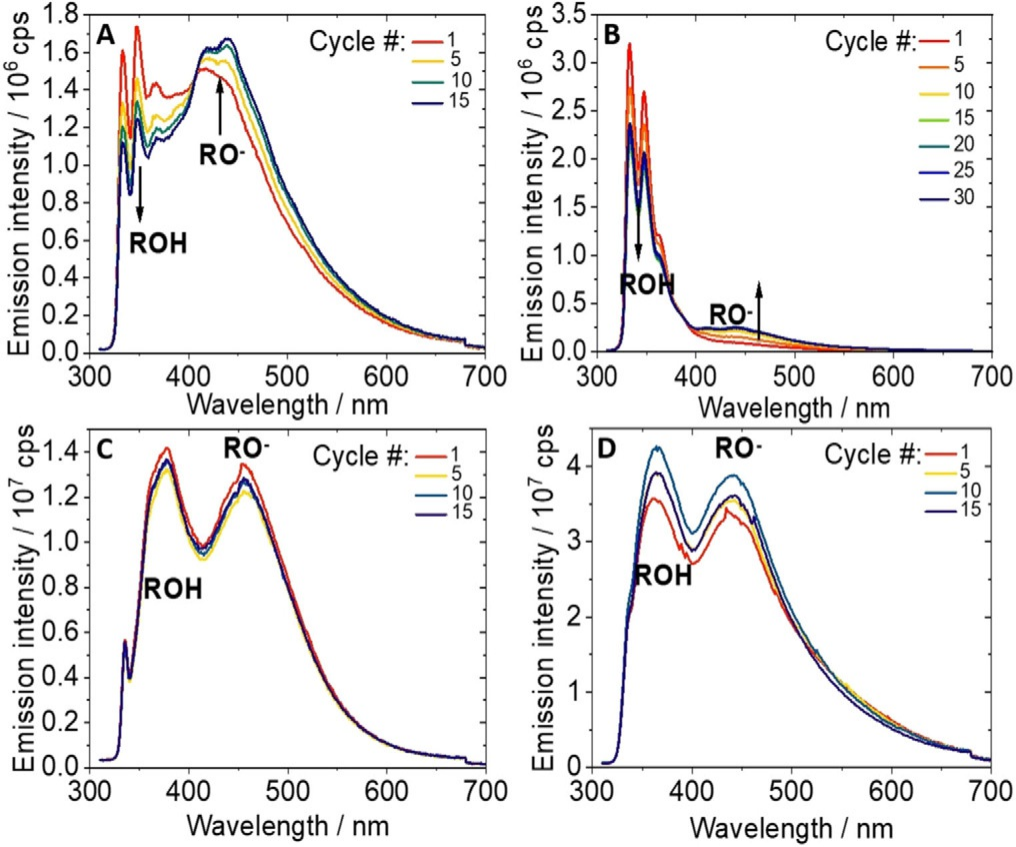
Emission spectra recorded under inert conditions with continuous irradiation at *λ*_exc_=300 nm of A) P[MEO_9_MA_*x*_ **‐** *co* **‐**NOeMAm_*y*_], B) P[MEO_9_MA_*x*_ **‐** *co* **‐**NOeMA_*y*_], C) P[MEO_9_MA_*x*_ **‐** *co* **‐**NMAm_*y*_], and D) P[MEO_9_MA_*x*_ **‐** *co* **‐**VN_*y*_] in H_2_O. Light exposure time per cycle=4.5 min, and a Xe lamp (ozone**‐**free 450 W xenon bulb) served as the illumination source.

The change in protonation state of the 1‐naphthol in the different copolymers under continuous illumination at *λ*_exc_=300 nm was monitored (*P*=0.21 mW, *I*=7 mW cm^−2^, see Figure 5) over several emission scans until no pronounced spectral changes were observed anymore. In the case of P[MEO_9_MA_*x*_‐*co*‐NOeMAm_*y*_] and P[MEO_9_MA_*x*_‐*co*‐NOeMA_*y*_], the emission at 350 nm is assigned to the protonated 1‐naphthol, while the deprotonated form, RO^−^, emits at 450 nm. As seen in Figure 5, continuous illumination of the photoacid copolymers leads to a slow decrease in the ROH emission, and a concerted increase in the band assigned to the RO^−^ form. In contrast, for P[MEO_9_MA_*x*_‐*co*‐NMAm_*y*_] and P[MEO_9_MA_*x*_‐*co*‐VN_*y*_] the steady‐state equilibrium between ROH and RO^−^ is already reached within the first emission measurement, that is, within 4.5 min, which corresponds to the experimental times needed to collect each emission spectrum under the given experimental conditions; as such, no further significant changes in the dual ROH/RO^−^ emission are observed in subsequent measurements. However, changes in the protonation state of NOeMA‐ and NOeMAm‐based photoacid copolymers under illumination indicate that the copolymers undergo morphological changes, which results in a higher degree of hydration of the 1‐naphthol units. This leads to a higher fraction of 1‐naphthol moieties being accessible to H_2_O and facilitates subsequent ESPT. Hence, 1‐naphthol might also serve as a probe for conformational changes within the copolymer strand, that is, the formation of domains of higher degrees of solvation. In the case of P[MEO_9_MA_*x*_‐*co*‐NMAm_*y*_] and P[MEO_9_MA_*x*_‐*co*‐VN_*y*_] featuring excited state acidity constants p*K*_a_*<2, the photo‐stationary condition is reached faster than in the other systems (p*K*_a_*>2 in P[MEO_9_MA_*x*_‐*co*‐NOeMA_*y*_] and P[MEO_9_MA_*x*_‐*co*‐NOeMAm_*y*_]).

### Reversibility of the deprotonation and photostability

We further investigated the reversibility of the 1‐naphthol deprotonation using P[MEO_9_MA_*x*_‐*co*‐NOeMAm_*y*_] as a benchmark since NOeMAm showed the most pronounced changes in its protonation state during UV illumination. To evaluate the reprotonation, two series of emission spectra were collected from a sample solution that was irradiated twice under identical conditions (deaerated, *λ*_exc_=300 nm, *P*_300 nm_=0.69 mW, *I*=12 mW cm^−2^) and kept in the dark between measurements (except for the emission measurements, seen in Figure S10A and B). Again, an increase in the RO^−^ emission is observed upon illumination at 300 nm until a steady state is reached. The reversibility of the process was evaluated by normalizing the first and last spectra obtained in each measurement series to the maximum emission of pure ROH ($\lambda_{\max}^{\mathrm{em}}=\;334\;\;nm$ , see Figure S10 D**)**. Comparison of the integrated emission ratio, ROH/RO^−^, of the 1^st^ cycle indicates that under these conditions the reprotonation of 1‐naphthol occurs only to a certain extent (see Table 3). UV/Vis spectra collected prior to and after UV illumination of P[MEO_9_MA_*x*_‐*co*‐NOeMAm_*y*_] confirm these results (Figure S10C). As 1‐naphthols (or in general hydroxyarenes) show a variety of photoreactions, for example, photooxidation,^44^ this irreversible behavior could indicate another photoreaction occurring in parallel to the ESPT, or is subsequent to the 1‐naphthol deprotonation. Decreasing the power of the illumination source to *P*_300 nm_=0.21 mW (*I*=7 mW cm^−2^, see Figure S11) leads to a distinct decrease in RO^−^ formation. Moreover, the stationary state condition of protonated to deprotonated 1‐naphthol is reached within less measurement cycles during the 2nd series, and the deprotonated form RO^−^ is generated to a large extent (ca. 70 %).

**Table 3:** Integrated emission ratio of protonated (ROH) and deprotonated (RO^‐^) 1‐naphthol in P[MEO_9_MA_*x*_‐*co*‐NOeMAm_*y*_]; cycles were performed until no significant spectral changes in emission were observed.

*I*=7 mW cm^−2^		*I*=12 mW cm^−2^	
Series (cycle)	Integrated emission ratio ROH/ RO^‐^	Series (cycle)	Integrated emission ratio ROH/RO^−^
1 (1)	1:1.45	1 (1)	1:1.95
1 (25)	1:3.16	1 (30)	1:4.38
2 (1)	1:2.01	2 (1)	1:2.57
2 (25)	1:3.57	2 (30)	1:4.80

**Figure 6:**
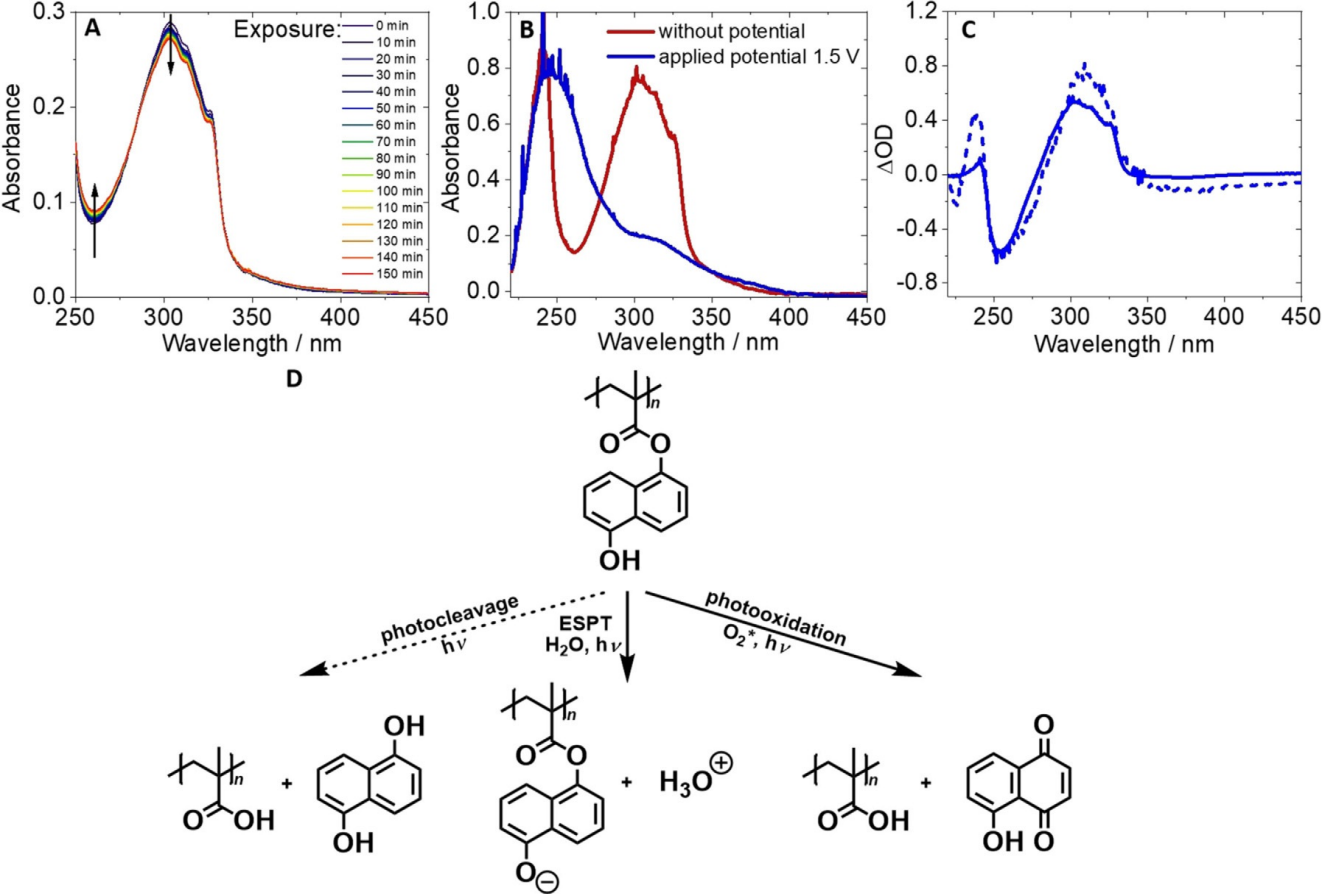
A) UV/Vis spectra of P[MEO_9_MA_*x*_ **‐** *co* **‐**NMA_*y*_] in aerated H_2_O during irradiation at 365 nm. B) UV/Vis spectra of P[MEO_9_MA_*x*_ **‐** *co* **‐**NMA_*y*_] in 0.1 m aqueous KCl, applied potential 0 V (red) and **+**1.5 V versus Ag/AgCl (blue). C) UV/Vis difference spectra of P[MEO_9_MA_*x*_ **‐** *co* **‐**NMA_*y*_] obtained in UV/Vis SCE measurement (solid lines) and UV/Vis irradiation studies (dashed lines). D) Proposed pathways after photoexcitation in H_2_O.

A fundamental property which determines the usability of a chromophore is its photostability. Besides earlier described investigations concerning photocleavage, the photostability of the 1‐naphthol containing copolymers was also investigated through steady‐state UV/Vis absorption in H_2_O under aerated conditions. Upon illumination at 365 nm, the main absorption band located at approximately 300 nm decreases, while in the region around 260 nm a slight increase in absorption is observed (see Figure 6 A). The degree of photodegradation was monitored as the relative change in absorbance for the main absorption band during UV light exposure and all investigated copolymers exhibited high photostability with degrees of photodegradation below 10 % (illumination for 2.5 h at 365 nm, *P*_365 nm_=85 mW) in O_2_‐saturated H_2_O.

UV/Vis spectroelectrochemistry (SEC)^45^ was conducted to investigate the absorption properties of the electrochemically oxidized 1‐naphthol species and was aimed to identify a possible oxidative degradation pathway during exposure to UV light (Figure 6 B). Under oxidative conditions, we observe an absorption maximum at 245 nm and a lowered absorbance in the range of 300 nm. In our opinion, these features qualitatively explain the changes in absorbance upon UV illumination: excitation of P[MEO_9_MA_*x*_‐*co*‐“N”_*y*_] can lead to three main pathways (Figure 6 C). As described above, ESPT occurs mainly for photoacid moieties that are accessible for H_2_O, which is accompanied by low degrees of photocleavage. In addition, a combination of photooxidation and potential photocleavage can occur, leading to the formation of a Juglone derivative.^46^ By assessment of the differential absorption spectra obtained in UV/Vis SEC and UV/Vis irradiation studies (see Figures 6 C and S12) we assign the spectral changes upon UV illumination to the photooxidative pathway. Nevertheless, a quantitative assessment to which extent photocleavage and photooxidation contribute to photoacid degradation occurring is not feasible, at least based on the optical spectroscopy used herein.

### Light‐triggered release of cargo from photoacid‐containing block copolymer micelles

**Figure 7:**
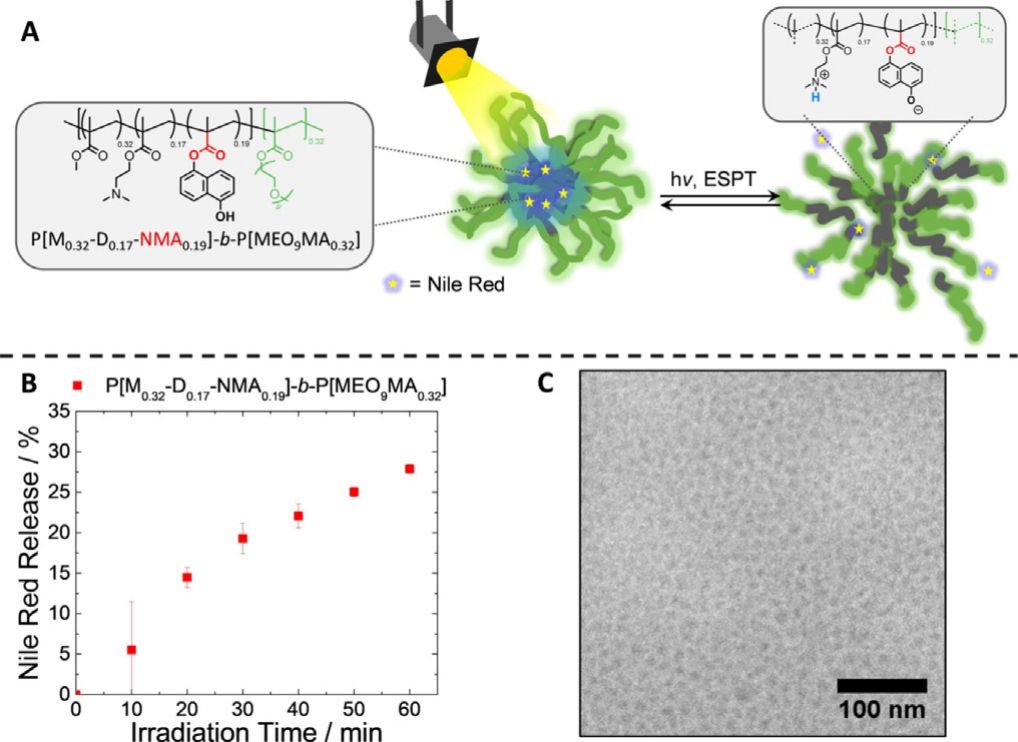
A) Scheme showing light**‐**triggered release of encapsulated NR from P[M_0.32_ **‐**D_0.17_ **‐**NMA_0.19_]**‐** *b* **‐**P[MEO_9_MA_0.32_] block copolymer micelles. B) NR release from P[M_0.32_ **‐**D_0.17_ **‐**NMA_0.19_]**‐** *b* **‐**P[MEO_9_MA_0.32_]. C) cryo**‐**TEM images of micelles prepared from P[M_0.32_ **‐**D_0.17_ **‐**NMA_0.19_]**‐** *b* **‐**P[MEO_9_MA_0.32_] before irradiation.

After evaluating the photophysical properties of photoacid‐containing water‐soluble copolymers in general, we wanted to go one step further and were interested in whether photoacids within amphiphilic block copolymers can be used to release encapsulated cargo upon irradiation. We therefore designed an amphiphilic block copolymer (BCP) as a representative example carrying the photoacid comonomer NMA in the hydrophobic block and featuring a hydrophilic block of MEO_9_MA. In aqueous media, these materials undergo self‐assembly into core‐corona micelles where P[MEO_9_MA] serves as corona and the NMA‐containing segment as core. More specifically, the hydrophobic block consists of a terpolymer comprising methyl methacrylate (MMA/“M”), 2‐(*N*,*N*‐dimethylamino)ethyl methacrylate (DMAEMA/“D), and NMA (Figure 7).

Upon irradiation with light, ESPT can occur whereby adjacent DMAEMA units act as a base and are protonated, shifting the overall hydrophilic/hydrophobic balance of the core‐forming block and this results in swelling or disruption of the micelles to enable cargo release. The underlying amphiphilic diblock quarterpolymer P[(M_33_‐D_11_‐NMA_13_)‐*b*‐(MEO_9_MA)_23_] was synthesized under comparable conditions as discussed earlier using RAFT polymerization, followed by subsequent deprotection. In this case, we targeted a 1:1 mass ratio between both blocks (Figure S13, the characterization data, both NMR and SEC, can be found in Table 4 as well as in the Supporting Information, Figure S14 and Figure S15).

**Table 4:** Characterization data (SEC, NMR) for the (*t*)NMA carrying (block) copolymers.

Polymer^[a]^	*M*_*n,*,SEC_^[b]^ [kg mol^−1^]	*Ð* ^[a]^	Conv^[c]^ [%]	*DP*_n,NMR_^[d]^ (block)	*M*_n,NMR_ [kg mol^−1^]	O^[e]^ [wt.%]
P[M_0.55_‐D_0.22_‐*t*NMA_0.23_]	14	1.09	77	58	10	–
P[M_0.32_‐D_0.17_‐*t*NMA_0.19_]‐*b*‐P[MEO_9_MA_0.32_]	21	1.14	54	23	21.5	53.5
P[M_0.32_‐D_0.17_‐NMA_0.19_]‐*b*‐P[MEO_9_MA_0.32_]	23.5	1.15	–	–	–	58.1

[a] The indices of the polymers depict the DPs of each monomer. [b] SEC (DMAc/LiCl) (PMMA calibration). [c] ^1^H NMR (300 MHz, CDCl_3_). [d] Determined from the conversion. [e] Weight fraction of the MEO_9_MA (abbreviated with O) block or hydrophilic content estimated from *M*_n,NMR_.

Micellar solutions from P[M_0.32_‐D_0.17_‐NMA_0.19_]‐*b*‐P[MEO_9_MA_0.32_] were prepared by first dissolving the diblock quaterpolymer in THF as a non‐selective solvent, followed by adding the solution dropwise into micropure water and allowing the THF to evaporate over 24 h. Nile red (NR) as a model hydrophobic cargo was encapsulated through co‐precipitation by using a certain amount of a NR stock solution in THF.

Since the fluorescence quantum yield of NR strongly depends on the polarity of the solvent, this can be taken as a measure for the swelling of the block copolymer micelles. Upon irradiation, a distinct decrease in emission intensity (Figure S16) hinting towards the release of NR is observed. Furthermore, with increasing irradiation time more pronounced changes are visible (Figure 7 B), which we interpret as the release of approximately 30 % of the Nile red within 60 minutes. The formation of well‐defined spherical micelles was confirmed by cryogenic transmission electron microscopy (cryo‐TEM, Figure 7 C). As can be seen, narrowly dispersed spherical micelles with core sizes of about 20 nm in diameter are formed and no significant difference in morphology or micellar size could be observed after irradiation (Figure S16C). The ability to effectively swell these micelles to release the encapsulated Nile red was further supported by a DLS experiment where we mimicked the photo‐induced deprotonated state of the photoacidic moiety by dialyzing the prepared micelles against a pH 10 buffer solution (Figure S17).

## Conclusions

The herein reported 1‐naphthol containing water soluble copolymers P[MEO_9_MA_*x*_‐*co*‐“N”_*y*_] are interesting materials for any application where temporally and spatially controlled release of protons is desirable. Besides design of well‐defined copolymers using controlled radical copolymerization (RAFT), we also investigated the ground and excited state acidity of the photoacids by means of steady‐state absorption and emission spectroscopy. Thereby, we could show that the local microenvironment of the 1‐naphthol, that is, presumably the accessibility of the 1‐naphthol units within the copolymer, strongly affects its tendency to undergo ESPT. Among the six different copolymers studied herein, P[MEO_9_MA_*x*_‐*co*‐NOeMA_*y*_] and P[MEO_9_MA_*x*_‐*co*‐NOeMAm_*y*_] were characterized by a less pronounced increase in acidity upon irradiation (p*K*_a_*>2), and in these cases we could monitor reversible deprotonation—also accompanied by proposed morphological changes upon irradiation (increased degree of hydration and, with that, improved accessibility of the photoacids for water molecules). Besides the desired ESPT, we also investigated the photo‐degradation of the herein described materials and could identify photo‐oxidation as a potential additional pathway upon illumination. Among the materials presented herein, VN‐based copolymers so far exhibit excellent photostability. In a final proof‐of‐concept experiment we demonstrated the incorporation of NMA into the core of block copolymer micelles and that this can be used to trigger light‐mediated release of encapsulated cargo. Although the exact mechanism and kinetics still demand further studies, we foresee great potential of photoacid‐containing polymeric materials for site‐ and time‐controlled release applications. The photoacid type and content incorporated may prove to be a facile way to tune the release properties beyond established protocols. We believe that the concept introduced here is of general interest in material science wherever (light‐mediated) polarity or charge gradients are desirable on surfaces or within multilayer materials.

## Experimental Section

All starting materials were purchased from Sigma–Aldrich (1‐hydroxy‐2‐naphthoic acid, ≥97.0 %; trimethylamine, ≥99 %; 1,4‐dioxane anhydrous, 99.8 %; 2,4,6‐trimethylpyridine, ≥99.0 %; 4‐(dimethylamino)pyridine ≥98.0 %; tetrabutylammonium fluoride trihydrate (TBAF), ≥97.0 %; 1,3,5‐trioxane, ≥99 %; 4,4′‐Azobis(4‐cyanovaleric acid) (V‐501, ≥98.0 %), Carbolution (di‐*tert*‐butyl dicarbonate, 99 %), ABCR (imidazole, 99 %; *tert*‐butyldimethylsilyl chloride, 98 %; *N*‐Boc‐ethylenediamine, 98 %; *N*‐ethyl‐*N*′‐(3‐dimethylaminopropyl)carbodiimide hydrochloride, EDC‐HCl, 98 %), J&K (*N*‐phenyl‐bis(trifluoromethanesufonimide), 99 %), Fluorochem Ltd. (Pd(PPh_3_)_4_, 98 %; vinyltri‐*n*‐butyltin, 95 %), TCI (3‐hydroxy‐1,2,3‐benzotriazin‐4(3*H*)‐one (HO‐Dhbt), >98.0 %), VWR (acetic acid, ≥99 %), Strem Chemicals (2‐cyano‐2‐propyl benzodithioate (CPDB), 97 %; 4‐cyanopentanoic acid dithiobenzoate (CPADB), ≥97.0 %) or Carl Roth (trifluoroacetic acid, ≥99,9 %; 2,2,2‐trifluorethanol, >99,8 %) were used as received if not mentioned otherwise. All deuterated solvents were obtained from Deutero. Nona(ethylene glycol) methyl ether methacrylate (MEO_9_MA or oligo(ethylene glycol) methyl ether methacrylate (OEGMA), *M_n_*=500 g mol^−1^), methyl methacrylate (MMA abbreviated with M herein, 99 %) and 2‐(*N*,*N*‐dimethylamino)ethyl methacrylate (DMAEMA abbreviated with D herein, 98 %) were purchased from Sigma–Aldrich and passed over a short column of either inhibitor remover from Sigma–Aldrich or aluminum oxide to remove the inhibitor prior to use. 2,2′‐Azobis(iso‐butyronitrile) (AIBN, Sigma–Aldrich) was recrystallized from ethanol and stored in the freezer. 4‐Dimethylaminopyridine (DMAP) was recrystallized from toluene. 1,5‐Dihydroxynaphthalene was recrystallized from water/ethanol (10:1 v/v). Pentafluorophenyl methacrylate (FMA),^47^ 2‐bromoethyl methacrylate,^48^ and *tert*‐butyl 2‐bromoethylcarbamate^49^ were synthesized according to literature procedures. 5‐{(*tert*‐Butyldimethylsilyl)oxy}naphthalen‐1‐ol (**1**), *t*NMA (**6**) and *t*NMAm (**7**) were synthesized as reported previously.^33^ *N*,*N*‐Dimethylformamide (DMF) and tetrahydrofuran (THF) were purified using a PureSolv‐EN solvent purification system (InnovativeTechnology). For preparative size exclusion chromatography, BioBeads™ SX‐1 column material from Bio‐Rad was used with THF as the eluent. All glassware was cleaned in a KOH/isopropanol bath and dried at 110 °C prior to use.

**Nuclear magnetic resonance (**NMR**) spectroscopy**: ^1^H NMR, ^13^C NMR, and ^19^F NMR spectra were measured on a 300 or 400 MHz Bruker AC spectrometer at 298 K using the residual solvent resonance as an internal standard. The chemical shifts are given in ppm.

**Size exclusion chromatography (SEC)**: SEC was performed on an Agilent 1200 series system equipped with a G1310A pump, a G1315D DA detector, a G1362A RI detector, and PSS GRAM 30 Å/1000 Å (10 μmol particle size, Polymer Standards Service GmbH, Mainz, Germany) columns in series at 40 °C using *N*,*N*‐dimethylacetamide (DMAc) with 2.1 g L^−1^ LiCl as eluent at a flow rate of 1 mL min^−1^. The system was calibrated with PMMA standards (*M*_p_=505 to 981 000 g mol^−1^).

**Elemental analyses (EA)**: Elemental analyses were performed on a HEKAtech EuroVector EA3000.

**Mass spectrometry**: For high resolution electron ionization mass spectrometry (HR‐EI‐MS) a Finnigan MAT95XL sector field mass spectrometer was used. For mass matching the signals were annotated relative to perfluoro kerosene signals nearby.

**Steady‐state UV/Vis and emission spectroscopy**: The steady‐state absorption spectra were measured in quartz cuvettes with 1 cm optical pathway (Hellma) using a Jasco V780 UV/Vis/NIR spectrophotometer. For UV/Vis photostability measurements the copolymers dissolved in O_2_‐saturated H_2_O were irradiated within the UV/Vis spectrometer using a 365 nm LED (Thorlabs, M365LP1, *P*_365 nm_=85 mW). The UV/Vis spectra under illumination were collected on a Jasco V530 UV/Vis spectrophotometer. Steady state emission measurements were carried out on a FLS980 emission spectrometer from Edinburgh Instruments using a Xe lamp (ozone free 450 W xenon bulb) as excitation source. For measurements under deaerated conditions the solvent (i.e., H_2_O) was deoxygenated by applying 4–5 freeze‐pump‐thaw cycles. UV/Vis SEC (spectroelectrochemistry) measurements were performed using a three‐electrode thin‐layer spectroelectrochemical cell with a pathlength of 1 mm (Hellma, Bioanalytical Systems, USA). The three‐electrode system contains a Pt counter electrode, an Ag/AgCl pseudo‐reference electrode and a glassy carbon working electrode. Potential controlled monitoring was performed using a computer controlled VersaSTAT 3 (Princeton Applied Research) potentiostat. UV/Vis spectra were recorded immediately after applying the potential to monitor the accompanied spectral changes. UV/Vis spectra were collected in transmission mode by using a product of Avantes Inc., which is comprised of a single‐channel fiber‐optic spectrometer (AvaSpec‐ULS2048XL) equipped with a deuterium‐halogen light source (AvaLight DH‐S‐BAL).

**Cryogenic transmission electron microscopy (cryo‐TEM)**: Cryogenic transmission electron microscopy (cryo‐TEM) measurements were performed on a FEI Tecnai G2 20 cryo‐transmission electron microscope (Jena Center for Soft Matter). Acceleration voltages were set to 200 kV. Samples were prepared on Quantifoil grids (3.5/1) after cleaning by an argon plasma treatment for 120 s. The sample solutions (10 μL) were blotted using a Vitrobot Mark IV. Samples were then plunge‐frozen in liquid ethane and stored under nitrogen before being transferred to the microscope using a Gatan transfer stage. TEM images were acquired with a 200 kV FEI Tecnai G2 20 equipped with a 4k *x* 4k Eagle HS CCD and a 1k × 1k Olympus MegaView camera.

**Dynamic light scattering (DLS)**: Dynamic light scattering (DLS) was performed using a custom‐built ALV/DLS‐90 set‐up, a ALV/CGS‐3 Goniometer system, equipped with a Cobolt Samba™ 532 nm single frequency CW diode pumped laser, an ALV/LSE‐5004 correlator, and a four quadrant detector. Measurements were recorded at an angle of 90 ° in UV transparent Macro Fluorescence cuvettes with 4 clear optical windows under ambient conditions. The particle size was determined using ALV‐Correlator Software V‐3.0 by applying a CONTIN fit. The custom‐built set‐up also allowed simultaneous in situ irradiation with a 365 nm Fiber‐Coupled LED (ThorLabs, M365FP1, 9.8 mW, 1400 mA).

**General procedure for the RAFT copolymerization**: Solutions containing the initiator (AIBN), CTA (CPDB), and monomer in 1,4‐dioxane were first prepared with a [M]:[CTA]:[I] ratio of 25:1:0.25 in a microwave vial. The total monomer concentration was adjusted to 2 m, or in the case of *t*NMAm and *t*VN, the copolymerizations were carried out in bulk. For kinetic investigations, 1,3,5‐trioxane was added as an internal standard, and samples were taken before and during the polymerization to determine the monomer conversion by ^1^H NMR spectroscopy in CDCl_3_. After sealing the reaction vessel with a suitable septum, the reaction mixture was deoxygenated by flushing with argon for 10 min. The solution polymerizations were carried out in an oil bath at 70 °C for 3 h. The bulk polymerizations were carried out in an oil bath at 70 °C for 24 h. The polymers were isolated through preparative size exclusion chromatography (Biobeads™ S‐X1) by using THF as eluent. The resulting copolymers were precipitated twice in *n*‐hexane and dried in vacuo.

P[MEO_9_MA_*x*_‐*co*‐*t*“N”_*y*_]: ^1^H NMR (300 MHz, CD_2_Cl_2_): *δ*=8.5–6.7 (aromatic), 4.5–4.3 (‐OCH_2_C*H*_2_O‐Naphthol, for *t*NOeMA), 4.3–4.0 (‐(OC*H*_2_CH_2_‐(EO)_8_‐), 3.7–3.4 (‐OCH_3_ and ‐(EO)_9_‐), 3.3 (‐(EO)_9_‐OC*H*_3_), 2.2–0.7 (backbone and Si(CH_3_)_2_C(C*H*_3_)_3_)), 0.3 ppm (‐Si(C*H*_3_)_2_C(CH_3_)_3_). P[MEO_9_MA_*x*_‐*co*‐PFMA_*y*_]: ^1^H NMR (400 MHz, CD_2_Cl_2_): *δ*=7.88, 7.54, and 7.38 (Ar‐H, CPADB), 4.3–4.0 (‐(OC*H*_2_CH_2_‐(EO)_8_‐), 3.7–3.4 (‐OCH_3_ and ‐(EO)_9_‐), 3.3 (‐(EO)_9_‐OC*H*_3_), 2.2–0.7 ppm (backbone) ppm. ^19^F NMR (400 MHz, CD_2_Cl_2_): *δ*=−162.84 (2F), −158.61 (1F), −149.94 ppm (2F). SEC (DMAc/LiCl, PMMA calibration) data is listed in Table 1.

**General procedure for the RAFT terpolymerization of MMA (M), DMAEMA (D) and** ***t*** **NMA**: The RAFT agent (CPADB), initiator (AIBN, 0.25 equiv. to RAFT agent), and monomers (125 equiv. to RAFT agent or 500 equiv. to P(O_20_) in case of block extension, M:D:*t*NMA=60:20:20) were weighed out into a microwave vial charged with a magnetic stirrer bar. The mixture was then diluted with 1,4‐dioxane to give a final monomer concentration of 4 M. For determination of the DP, 1,3,5‐trioxane was added as an internal standard, and samples were taken before and after the terpolymerization to determine the monomer conversion by ^1^H NMR spectroscopy in CDCl_3_. After sealing the reaction vessel with a suitable septum, the reaction mixture was deoxygenated by flushing with argon for 10 min. The terpolymerization was then initiated by placing the flask into a thermostatted oil bath pre‐heated to 70 °C. After eight hours, the terpolymerization was quenched by freezing in liquid nitrogen and exposure to air. The reaction mixture was then diluted with dichloromethane and precipitated into a 1:1 (v/v) mixture of *n*‐hexane and diethyl ether 3 times before being dried in vacuo.

P(M_0.55_‐D_0.22_‐*t*NMA_0.23_): ^1^H NMR (300 MHz, CD_2_Cl_2_): *δ*=8.5–6.7 (aromatic), 4.3–3.9 (‐OC*H*_2_CH_2_NH(CH_3_)_2_), 3.7–3.3 (‐OCH_3_), 2.7–2.5 (‐OCH_2_C*H*_2_NH(CH_3_)_2_), 2.5–0.7 (backbone and Si(CH_3_)_2_C(C*H*_3_)_3_)), 0.3 ppm (‐Si(C*H*_3_)_2_C(CH_3_)_3_). SEC (DMAc/LiCl, PMMA calibration) data can be found in Table 4.

**General block extension procedure with MEO_9_MA**: P(M_33_‐D_11_‐*t*NMA_13_), monomer (MEO_9_MA), and stock solution containing the initiator (V‐501, 0.25 equiv. to macro‐RAFT) were first measured out into a microwave vial charged with a magnetic stirrer bar. The reaction mixture was then further diluted with 1,4‐dioxane to give the desired monomer/solvent ratio of 1:1 (v/v) before being sealed with an aluminum cap fitted with a PTFE‐faced silicone septum. The reaction mixture was then deoxygenated by purging with argon for 20 min before being placed into a thermostatted oil bath preheated to 70 °C to initiate the polymerization. The polymerization was quenched after 90 min by cooling in liquid nitrogen and exposure to air. The reaction mixture was then diluted with dichloromethane and precipitated into a 1:1 (v/v) mixture of *n*‐hexane and diethyl ether 3 times before being dried in vacuo to yield P[M_0.32_‐D_0.17_‐*t*NMA_0.19_]‐*b*‐P[MEO_9_MA_0.32_] as a pink solid.

P[M_0.32_‐D_0.17_‐*t*NMA_0.19_]‐*b*‐P[MEO_9_MA_0.32_]: ^1^H NMR (300 MHz, CD_2_Cl_2_): *δ*=8.5–6.7 (aromatic), 4.3–3.9 (‐OC*H*_2_CH_2_NH(CH_3_)_2_ and ‐(OC*H*_2_CH_2_‐(EO)_8_‐), 3.7–3.3 (‐OCH_3_ and ‐(EO)_9_‐), 3.3 (‐(EO)_9_‐OC*H*_3_), 2.7–2.5 (‐OCH_2_C*H*_2_NH(CH_3_)_2_), 2.5–0.7 (backbone and Si(CH_3_)_2_C(C*H*_3_)_3_)), 0.3 ppm (‐Si(C*H*_3_)_2_C(CH_3_)_3_). SEC (DMAc/LiCl, PMMA calibration) data can be found in Table 4.

**Deprotection of the prepared copolymers and the diblock quarterpolymers**: The respective copolymers were dissolved in THF (*c*=0.1 g mL^−1^) and cooled to −20 °C before being deoxygenated by purging with Ar for 10 min. A deoxygenated solution of TBAF and acetic acid (respectively 2 equiv. with respect to the *t*“N” units) in THF was then added, and the solutions stirred for 3 h, warming to room temperature during this time. The copolymers were isolated by preparative size exclusion chromatography (Biobeads™ S‐X1) using THF as eluent. Subsequently, the resulting copolymers were precipitated twice in *n*‐hexane before being dried in vacuo.

P[MEO_9_MA_*x*_‐*co*‐“N”_*y*_]: ^1^H NMR (300 MHz, CD_2_Cl_2_): *δ*=8.5–6.7 (aromatic), 4.5–4.3 (‐OCH_2_C*H*_2_O‐Naphthol, for *t*NOeMA), 4.3–4.0 (‐(OC*H*_2_CH_2_‐(EO)_8_‐, 3.7–3.4 (‐OCH_3_ and ‐(EO)_9_‐), 3.3 (‐(EO)_9_‐OC*H*_3_), 2.2–0.7 ppm (backbone). SEC (DMAc/LiCl, PMMA calibration) data is listed in Table 1. P[M_0.32_‐D_0.17_‐NMA_0.19_]‐*b*‐P[MEO_9_MA_0.32_]: ^1^H NMR (300 MHz, CD_2_Cl_2_): *δ*=8.3–6.7 (aromatic), 4.4–3.9 (‐OC*H*_2_CH_2_NH(CH_3_)_2_ and ‐(OC*H*_2_CH_2_‐(EO)_8_‐), 3.7–3.4 (‐OCH_3_ and ‐(EO)_9_‐), 3.3 (‐(EO)_9_‐OC*H*_3_), 2.8–2.4 (‐OCH_2_C*H*_2_NH(CH_3_)_2_), 2.4–0.7 ppm (backbone and Si(CH_3_)_2_C(C*H*_3_)_3_)). SEC (DMAc/LiCl, PMMA calibration) for P[(M_33_‐D_11_‐NMA_13_)‐*b*‐(MEO_9_MA)_23_] can be found in Table 4.

**Post‐polymerization modification of P[MEO_9_MA**_***x***_ **‐*co*‐PFMA**_***y***_ **]**: The synthetic procedure was adapted and modified from a literature recipe.^38^ The respective copolymers were mixed together with 5 equiv. (with respect to the FMA units) of the amines **10** or **11**, 3‐hydroxy‐1,2,3‐benzotriazin‐4(3*H*)‐one (HO‐Dhbt) and trimethylamine. Subsequently, the mixture was dissolved in DMF. Afterwards, the solution was deoxygenated by purging with Ar for 10 min before being heated to 70 °C for 48 h. The solvent was then evaporated in vacuo, and the copolymers isolated by preparative size exclusion chromatography (Biobeads™ S‐X1) using THF as eluent. The resulting copolymers were then precipitated twice in *n*‐hexane and dried in vacuo.

P[MEO_9_MA_*x*_‐*co*‐“N”_*y*_]: ^1^H NMR (400 MHz, CD_2_Cl_2_): *δ*=8.7–6.5 (aromatic), 4.5–4.3 (‐NH‐CH_2_C*H*_2_‐X‐Naphthol), 4.3–3.9 (‐(OC*H*_2_CH_2_‐(EO)_8_‐), 3.7–3.4 (‐OCH_3_ and ‐(EO)_9_‐), 3.3 (‐(EO)_9_‐OC*H*_3_), 2.2–0.7 ppm (backbone). SEC (DMAc/LiCl, PMMA calibration) data is listed in Table 1.

**Micelle preparation**: For micelle preparation, 10 mg of the diblock quaterpolymer were dissolved in 1 mL of THF. The solution was added dropwise to 10 mL of micropure water in a glass vial under stirring. Stirring was continued in an open vial for 24 h to allow THF to evaporate. Subsequently, the concentration was adjusted to 1 mg mL^−1^ by refilling the vial with micropure water prior to further investigation.

**Nile red encapsulation and release**: Nile red was encapsulated into the diblock quaterpolymer micelles through co‐precipitation. Briefly, 6 mg of the respective diblock quaterpolymer was dissolved in THF (6 mL). Then, 71.4 μL of a Nile red stock solution (*c*=0.14 mg mL^−1^) in THF was added. Under vigorous stirring, 12 mL of deionized water was added dropwise. The volatiles (THF) were allowed to evaporate under continuous stirring for 24 before the suspension was diluted with 48 mL of deionized water (*c*_block copolymer_=0.1 mg mL^−1^, *c*_Nile red_=2×10^−4^ mg mL^−1^). For the investigation of light‐mediated release of Nile red, the corresponding micellar solutions were directly irradiated using a Thorlabs LED M365LP1 (365 nm, 1150 mW, 17.6 μW mm^−2^, 1400 mA, equipped with a COP1‐A—Collimation Adapter, distance ≈10 cm) at different irradiation times up to 60 min. After every 10 min of UV light irradiation, a fluorescence emission spectrum was recorded between 525 and 750 nm using an excitation wavelength of 510 nm. The release of Nile red was calculated according to Equation (2):

(2) $$\mathrm{Release}\left(\%\right)=\frac{I_{o}-I_{t}}{I_{o}}\times 100$$

$I_{o}$ corresponds to the initial fluorescence intensity of Nile red at the emission maximum (approximately 630 nm), and $I_{t}$ corresponds to the fluorescence intensity of Nile red at time *t*.

Supporting information available: experimental details for monomer and copolymer synthesis, data on polymerization kinetics, UV/Vis data for monomers and copolymers.

## Supporting information

As a service to our authors and readers, this journal provides supporting information supplied by the authors. Such materials are peer reviewed and may be re‐organized for online delivery, but are not copy‐edited or typeset. Technical support issues arising from supporting information (other than missing files) should be addressed to the authors.

Supplementary
