# Supplementary material for: Polymeric Photoacids Based on Naphthols—Design Criteria, Photostability, and Light‐Mediated Release
Source: Chemistry. 2020 Jan 21;26(11):2365–79. doi: 10.1002/chem.201903819 (PMC7064900; doi:10.1002/chem.201903819)
Supplement: Supplementary file 1 — Supplementary [file CHEM-26-2365-s001.pdf]

CHEMISTRY
A European Journal

Supporting Information

Polymeric Photoacids Based on Naphthols—Design Criteria, Photostability, and Light-Mediated Release

Felix Wendler+,[a, b] Maria Sittig+,[b, c, d] Jessica C. Tom,[a, b] Benjamin Dietzek,*[b, c, d] and Felix H. Schacher*[a, b]

chem_201903819_sm_miscellaneous_information.pdf

Synthesis of monomers and functional amines

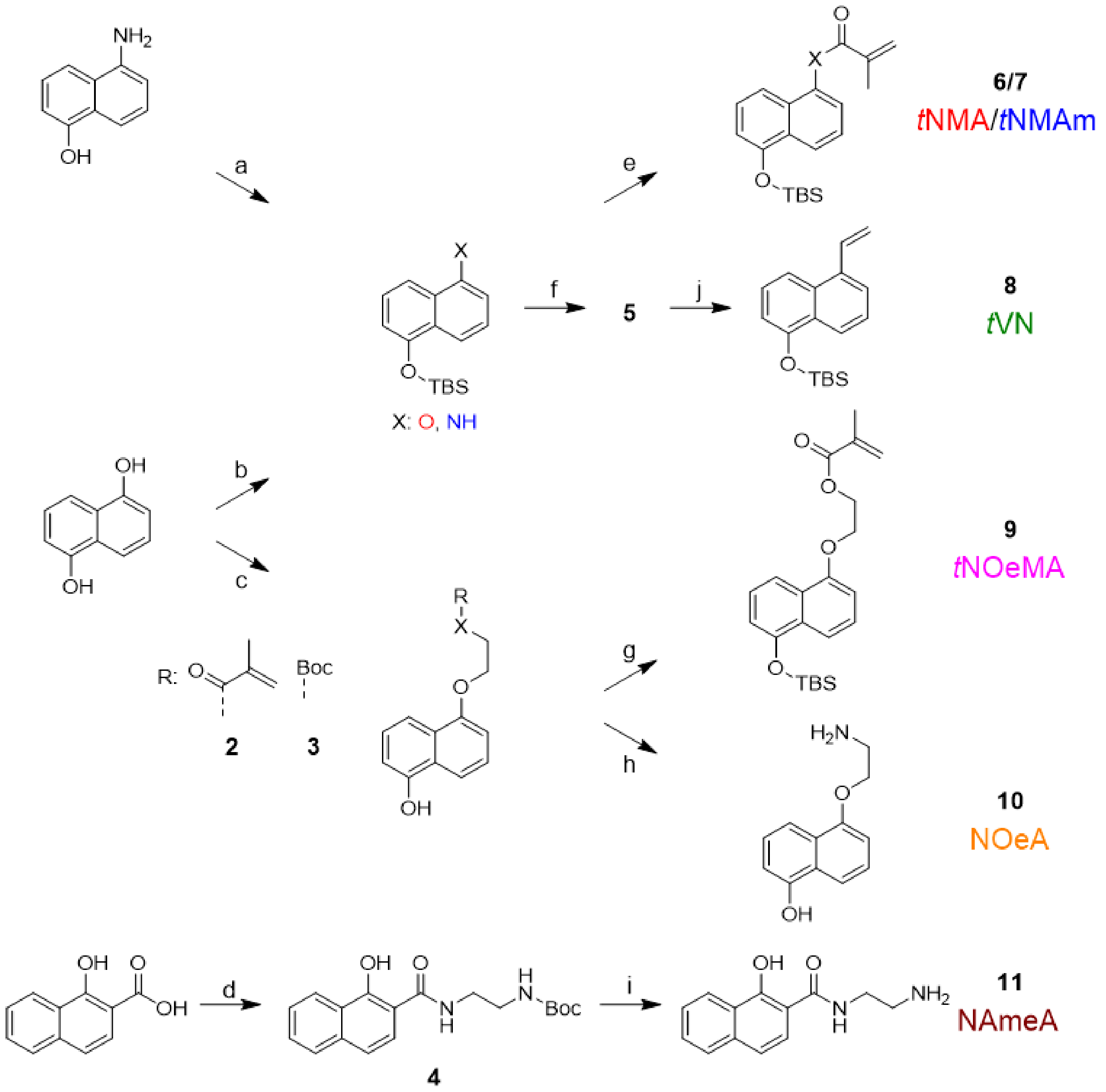

Figure S1: a) TBS-Cl, imidazole, THF, rt, 24 h, 69 % b) TBS-Cl, imidazole, THF, rt, 24 h, 47 %, c) K_2CO_3, DMF, rt, 12 h, 31 %/24 %, d) EDC-HCl, DMAP, DMF, rt, 12 h, 72 %, e) methacryloyl chloride, TEA, DCM, rt, 12 h, tNMA: 59 %, tNMAm: 39 %, f) X = O, Tf_2NPh, 2,4,6-trimethylpyridine, DMAP, THF, rt, 12 h, 81 %, g) TBS-Cl, imidazole, THF, rt, 12 h, 85 %, h) TFA, DCM, rt, 1 h, 66 %, i) TFA, DCM, rt, 1 h, 40 %, j) Vinyltri-*n*-butyltin, $Pd(PPh_3)_4$, LiCl, DMF, 80 °C, 12 h, 94 %.

Synthesis of 2-((5-hydroxynaphthalen-1-yl)oxy)ethyl methacrylate (NOeMA, 2) and BOC-(2-((5-hydroxynaphthalen-1-yl)oxy)ethyl)amine (BOC-NOeA, 3)

Under an Ar atmosphere, 1,5-dihydroxynaphthalene (1 eq.) and either 2-bromoethyl methacrylate or *tert*-butyl 2-bromoethylcarbamate (0.6 eq.) dissolved in DMF were added to K_2CO_3 (5 eq.). The reaction mixture was stirred at rt overnight. Afterwards, deionized water was added before being extracted 3 times with MTBE. The collected organic phases were dried over anhydrous $MgSO_4$ and the solvent removed under reduced pressure. The crude products were purified *via* column chromatography (silica

gel 60; NOeMA: ethyl acetate:*n*-hexane 1:3, yellowish brown solid, yield: 31 %; BOC-NOeA: ethyl acetate:*n*-hexane 1:3 + 5 % Et_3N, reddish solid, yield: 24 %).

NOeMA: ^{1}H-NMR (300 MHz, *d₆*-DMSO, *δ*): 10.09 (s, 1 H, -OH), 7.73 (d, 1 H, H-C_{ar}), 7.58 (d, 1 H, H C_{ar}), 7.30 (m, 2 H, H-C_{ar}), 6.93 (m, 2 H, H-C_{ar}), 6.06 (s, 1 H, =CH), 5.67 (s, 1 H, =CH), 4.58 (t, 2 H, -CH_2-CH_2-O-CO), 4.36 (t, 2 H, C_{ar}-O-CH_2-CH_2-), 1.89 (s, 3 H, -CH_3) ppm.

^{13}C-NMR (300 MHz, d_6-DMSO, *δ*): 166.56 (C=O), 153.66 (C_{ar}), 153.10 (C_{ar}), 135.07 (*C*=CH_2), 125.91 (C=*C*H_2), 125.71 (C_{ar}), 124.50 (C_{ar}), 114.64 (C_{ar}), 112.04 (C_{ar}), 108.79 (C_{ar}), 105.82 (C_{ar}), 66.27 (CH_2-*C*H_2-O-CO), 62.89 (C_{ar}-O-*C*H_2-CH_2-), 17.95 (-CH_3) ppm.

BOC-NOeA: ^{1}H-NMR (300 MHz, d_6-DMSO, *δ*): 10.02 (s, 1 H, -OH), 7.67 (d, 2 H, H-C_{ar}), 7.30 (t, 1 H, H-C_{ar}), 7.23 (t, 1 H, H-C_{ar}), 7.10 (t, 1H, -NH-Boc), 6.88 (t, 2 H, H-C_{ar}), 4.06 (t, 2 H, C_{ar}-O-CH_2-CH_2-), 3.43 (t, 2 H, -CH_2-CH_2-NH-Boc), 1.39 (s, 3 H, -CH_3) ppm.

^{13}C-NMR (300 MHz, d_6-DMSO, *δ*): 155.81 (C=O), 153.94 (C_{ar}-O-CH_2), 152.96 (C_{ar}-OH), 126.39 (C_{ar}), 125.53 (C_{ar}), 125.41 (C_{ar}), 124.60 (C_{ar}), 114.21 (C_{ar}), 112.60 (C_{ar}), 108.73 (C_{ar}), 105.20 (C_{ar}), 77.74 (C_{ar} O-*C*H_2-CH_2-O-CO), 67.05 (C_{ar}-O-CH_2-*C*H_2-O-CO), 28.26 (-CH_3) ppm.

Synthesis of BOC-1-hydroxynaphthalen-2-amido)ethyl)amine (BOC-NAmeA, 4)
1-Hydroxy-2-naphthoic acid (5.0 g, 26.57 mmol), *N*-Boc-ethylenediamine (1.5 eq., 6.4 g, 39.86 mmol), EDC-HCl, (1.5 eq., 7.6 g, 39.86 mmol) were dissolved in DMF (100 mL). 4-Dimethylaminopyridine (DMAP, 10 mol%, 0.3 g, 2.66 mmol) in DMF (2 mL) was added dropwise, and stirring was carried out overnight at room temperature. Deionized water was added and the respective mixture was extracted with MTBE thrice. The combined organic extracts were washed with water twice and then brine before being dried over anhydrous $MgSO_4$, filtered, and the solvent removed under reduced pressure. Subsequently, the crude product was purified by recrystallization from cyclohexane to obtain *tert*-butyl (2-(1-hydroxy-2-naphthamido)ethyl)carbamate as a white yellowish solid (6.3 g, 72 %).

^{1}H-NMR (300 MHz, d_6-DMSO, *δ*): 14.56 (s, 1 H, -OH), 8.99 (t, 1H, C_{ar}-CO-NH-), 8.24 (d, 1H, H-C_{ar}), 7.85 (t, 2H, H-C_{ar}), 7.63 (t, 1H, H-C_{ar}), 7.53 (t, 1H, H-C_{ar}), 7.37 (d, 1H, H-C_{ar}), 6.99 (t, 1H, -NH-Boc), 3.36 (q, 2H, C_{ar}-CO-NH-CH_2-CH_2-), 3.17 (q, 2H, -CH_2-CH_2-NH-Boc), 1.36 (s, 9 H, $C(CH_3)_3$) ppm.

^{13}C-NMR (300 MHz, d6-DMSO, *δ*): 170.4 (C=O), 159.62 (C_{ar}), 155.77 (C=O Boc), 135.75 (C_{ar}), 128.73 (C_{ar}), 127.39 (C_{ar}), 125.66 (C_{ar}), 124.72 (C_{ar}), 122.97 (C_{ar}), 122.69 (C_{ar}), 117.37 (C_{ar}), 107.07 (C_{ar}), 77.71 (-CH_2-*C*H_2-NH-Boc), 28.18 (-CH_3), ppm.

Synthesis of 5-((*tert*-butyldimethylsilyl)oxy)naphthalen-1-yl trifluoromethanesulfonate (*t*N-Tf, 5)
Under an Ar atmosphere, 2,4,6-trimethylpyridine (2 eq., 13.5 g, 111.50 mmol) and DMAP (0.1 eq., 0.7 g, 5.58 mmol) dissolved in THF was added to a solution of 5-((*tert*-butyldimethylsilyl)oxy)naphthalen-1-ol (15.3 g, 55.75 mmol) and *N*-phenyl-bis(trifluoromethanesufonimide) (1.5 eq., 29.9 g, 83.63 mmol) in THF. The reaction mixture was then

stirred at room temperature overnight. Subsequently, the THF was removed under reduced pressure and the residue re-dissolved in MTBE. After three washing steps using deionized water, the organic phase was dried over anhydrous $MgSO_4$, filtered, and the solvent removed under reduced pressure. The crude product was purified *via* column chromatography (silica gel 60; ethyl acetate:*n*-hexane 1:10, 18.3 g of a white solid, yield: 81 %).

^{1}H-NMR (300 MHz, CD_2Cl_2, δ): 8.28 (t, 1 H, H–C_{ar}), 7.66 (d, 1 H, H–C_{ar}), 7.48 (m, 3 H, H–C_{ar}), 7.03 (d, 1 H, H–C_{ar}), 1.11 (s, 9 H, $SiCCH_3$), 0.33 (s, 6 H, $SiCH_3$) ppm.

^{13}C-NMR (400 MHz, CD_2Cl_2, δ): 152.60 (C_{ar}), 146.27 (C_{ar}), 130.25 (C_{ar}), 128.83 (C_{ar}), 128.50 (C_{ar}), 124.77 (C_{ar}), 124.05 (C_{ar}), 118.78 (C_{ar}), 117.79 (CF_3), 114.56 (C_{ar}), 113.87 (C_{ar}), 26.16 (SiC*C*H_3), 18.91 (Si*C*CH_3), −4.53 ($SiCH_3$) ppm.

^{19}F-NMR (400 MHz, CD_2Cl_2, δ): -73.83 (-CF_3) ppm.

Synthesis of *tert*-butyldimethyl((5-vinylnaphthalen-1-yl)oxy)silane (*t*VN, 8)

Under an Ar atmosphere **5** (15.0 g, 36.90 mmol) together with 0.1 eq. of $Pd(PPh_3)_4$ (4,3 g, 3.69 mmol) and 0.02 eq. LiCl (31,3 mg, 0.74 mmol) were dissolved in dry DMF deoxygenated with Ar. Subsequently, vinyltri-*n*-butyltin (1.2 eq., 14.8 g, 44.3 mmol) deoxygenated with Ar was added dropwise. The mixture was then heated to 80 °C and stirred overnight. Deionized water was then added, and the respective mixture was extracted with MTBE. The combined organic extracts were washed with water thrice; dried over anhydrous $MgSO_4$, filtered, and the solvent removed under reduced pressure. Finally, the crude product was purified *via* column chromatography (silica gel 60; ethyl acetate:*n*-hexane 1:5, 9.9 g of a white solid, yield: 94 %).

^{1}H-NMR (300 MHz, CD_2Cl_2, δ): 8.22 (d, 1 H, H–C_{ar}), 7.74 (d, 1 H, H–C_{ar}), 7.66 (d, 1 H, H–C_{ar}), 7.45 (m, 2 H, H–C_{ar}), 7.41 (dd, 1 H, C*H*=CH_2), 6.96 (d, 1 H, H–C_{ar}), 5.84 (dd, 1 H, trans CH=C*H*$_2$), 5.51 (dd, 1 H, cis CH=C*H*$_2$), 1.14 (s, 9 H, $SiCCH_3$), 0.33 (s, 6 H, $SiCH_3$) ppm.

^{13}C-NMR (300 MHz, CD_2Cl_2, δ): 152.62 (C_{ar}), 135.92 (*C*H=CH_2), 135.30 (C_{ar}), 133.26 (C_{ar}), 128.75 (C_{ar}), 126.67 (C_{ar}), 125.42 (C_{ar}), 124.50 (C_{ar}), 123.25 (C_{ar}), 117.28 (CH=*C*H_2), 117.22 (C_{ar}), 113.32 (C_{ar}), 26.16 (SiC*C*H_3), 18.91 (Si*C*CH_3), 4.53 ($SiCH_3$) ppm.

HR-EI-MS (m/z): $[M]^+$ calcd for $C_{18}H_{24}OSi$, 284.1596; found, 284.1598.

Elemental analysis: calcd C: 76.00 %, H: 8.50 %; found: C: 76.27 %, H: 8.54 %.

Synthesis of 2-((5-((*tert*-butyldimethylsilyl)oxy)naphthalen-1-yl)oxy)ethyl methacrylate (*t*NOeMA, 9)

2 (6.0 g, 22.03 mmol) and *tert*-butyldimethylsilyl chloride (1.5 eq., 5,0 g, 33.05 mmol) were dissolved in THF before being deoxygenated with Ar. A deoxygenated solution of imidazole (1.5 eq., 2.25 g, 33.05 mmol) in THF was then added dropwise. The reaction mixture was then stirred at r.t. for 24 h. After filtration, the solvent was completely evaporated, and MTBE was added to the resulting residue.

Repeated extractions with deionized water were carried out, and the organic phase collected and dried over anhydrous $MgSO_4$ before the solvent was removed under reduced pressure. The crude product was then purified *via* column chromatography (silica gel 60; ethyl acetate:*n*-hexane 1:10, white solid, yield: 85 %).

^{1}H-NMR (300 MHz, d_6-DMSO, δ): 7.74 (d, 1 H, H-C_{ar}), 7.66 (d, 1 H, H-C_{ar}), 7.39 (t, 1 H, H-C_{ar}), 7.33 (t, 1 H, H-C_{ar}), 6.99 (d, 1 H, H-C_{ar}), 6.93 (d, 1 H, H-C_{ar}), 6.05 (s, 1 H, =CH), 5.66 (s, 1 H, =CH), 4.58 (t, 2H, -CH_2-CH_2-O-CO), 4.38 (t, 2H, C_{ar}-O-CH_2-CH_2-), 1.88 (s, 3 H, -CH_3), 1.01 (s, 9 H, $SiCCH_3$), 0.18 (s, 6 H, $SiCH_3$) ppm.

^{13}C-NMR (300 MHz, d_6-DMSO, δ): 166.47 (C=O), 153.79 (C_{ar}), 150.78 (C_{ar}), 135.71 (*C*=CH_2), 128.28 (C=*C*H_2), 126.28 (C_{ar}), 125.84 (C_{ar}), 125.35 (C_{ar}), 114.53 (C_{ar}), 114.48 (C_{ar}), 114.39 (C_{ar}), 113.33 (C_{ar}), 105.95 (C_{ar}), 66.33 (-CH_2-*C*H_2-O-CO), 62.82 (C_{ar}-O-*C*H_2-CH_2-), 25.61 (SiC*C*H_3), 18.02 (Si*C*CH_3), 17.90 (-CH_3), 4.55 ($SiCH_3$) ppm.

HR-EI-MS (m/z): $[M]^+$ calcd for $C_{22}H_{30}O_4Si$, 386,1913; found, 386,1905.

Elemental analysis: calcd C: 68.36 %, H: 7.82 %; found: C: 68.89 %, H: 7.85 %.

Synthesis of (2-((5-hydroxynaphthalen-1-yl)oxy)ethyl)amine (NOeA, 10) and (1-hydroxynaphthalen-2-amido)ethyl)amine (NAmeA, 11)

Trifluoroacetic acid (TFA, 10 eq.) dissolved in DCM was added dropwise to a solution of BOC-NOeA (**3**) or BOC-NAmeA (**4**) in DCM. After 1 h, excess DCM and TFA were removed under reduced pressure before the residue was re-dissolved in deionized water, neutralized by the addition of 0.1 M NaOH solution, and finally adjusting the pH to approximately 9. The aqueous phase was then extracted with ethyl acetate. The combined organic layers were washed with deionized water, dried over anhydrous $MgSO_4$, filtered, and the solvent removed under reduced pressure. Subsequently, the crude product was purified through recrystallization (**NOeA**: MeOH to obtain a reddish solid with a 66 % yield; **NAmeA**: THF to obtain a white-pinkish solid with a 40 % yield).

NOeA: ^{1}H-NMR (300 MHz, CD_3OD, δ): 7.78 (d, 1 H, H-C_{ar}), 7.72 (d, 1 H, H-C_{ar}), 7.24 (q, 2 H, H-C_{ar}), 6.86 (d, 1 H, H-C_{ar}), 6.78 (d, 1 H, H-C_{ar}), 4.02 (t, 2 H, C_{ar}-O-CH_2-CH_2-), 3.04 (t, 2 H, -CH_2-CH_2-NH_2) ppm.

^{13}C-NMR (300 MHz, CD_3OD, δ): 155.57 (C_{ar}), 154.45 (C_{ar}), 128.31 (C_{ar}), 127.45 (C_{ar}), 126.45 (C_{ar}), 125.48 (C_{ar}), 115.74 (C_{ar}), 113.86 (C_{ar}), 109.72 (C_{ar}), 106.28 (C_{ar}), 70.50 (C_{ar}-O-*C*H_2-), 41.87 (-CH_2-NH_2) ppm.

HR-EI-MS (m/z): $[M]^+$ calcd for $C_{12}H_{13}NO_2$, 203,0946; found, 203,0947.

Elemental analysis: calcd C: 70.92 %, H: 6.45 %, N: 6.89 %; found: C: 68.87 %, H: 6.33 %, N: 6.35 %.

NAmeA: ^{1}H-NMR (300 MHz, CD_3OD, δ): 8.38 (d, 1H, H-C_{ar}), 7.79 (d, 1H, H-C_{ar}),7.66 (d, 1H, H-C_{ar}), 7.47 (t, 1H, H-C_{ar}), 7.38 (t, 1H, H-C_{ar}), 7.05 (d, 1H, H-C_{ar}), 3.63 (t, 2H, C_{ar}-CO-NH-$\mathit{C}H_2$-CH_2-), 3.05 (t, 2H, -CH_2-CH_2-NH_2) ppm.

^{13}C-NMR (300 MHz, CD_3OD, δ): 173.42 (C=O), 166.40 (C_{ar}), 138.48 (C_{ar}), 130.00 (C_{ar}), 128.90 (C_{ar}), 128.22 (C_{ar}), 125.71 (C_{ar}), 125.24 (C_{ar}), 115.53 (C_{ar}), 110.13 (C_{ar}), 41.92 (C_{ar}-CO-NH-$\mathit{C}H_2$-CH_2-), 40.24 (-CH_2-$\mathit{C}H_2$-NH_2) ppm.

HR-EI-MS (m/z): $[M]^+$ calcd for $C_{13}H_{14}N_2O_2$, 230,1055; found, 230,1055.

Elemental analysis: calcd C: 67.81 %, H: 6.13 %, N: 12.17 %; found: C: 65.23 %, H: 6.82 %, N: 10.17 %.

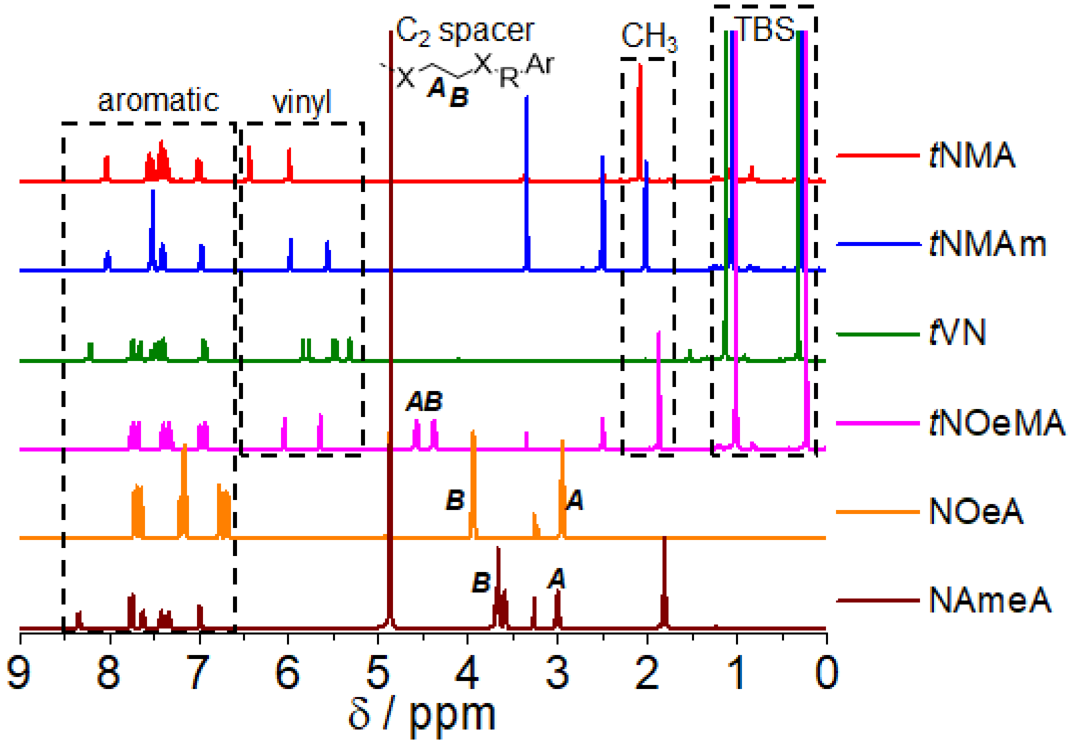

Figure S2: ^{1}H-NMR spectra of *t*NMA, *t*NMAm, *t*NOeMA, *t*VN in CD_2Cl_2, and NOeA, NAmeA in CD_3OD.

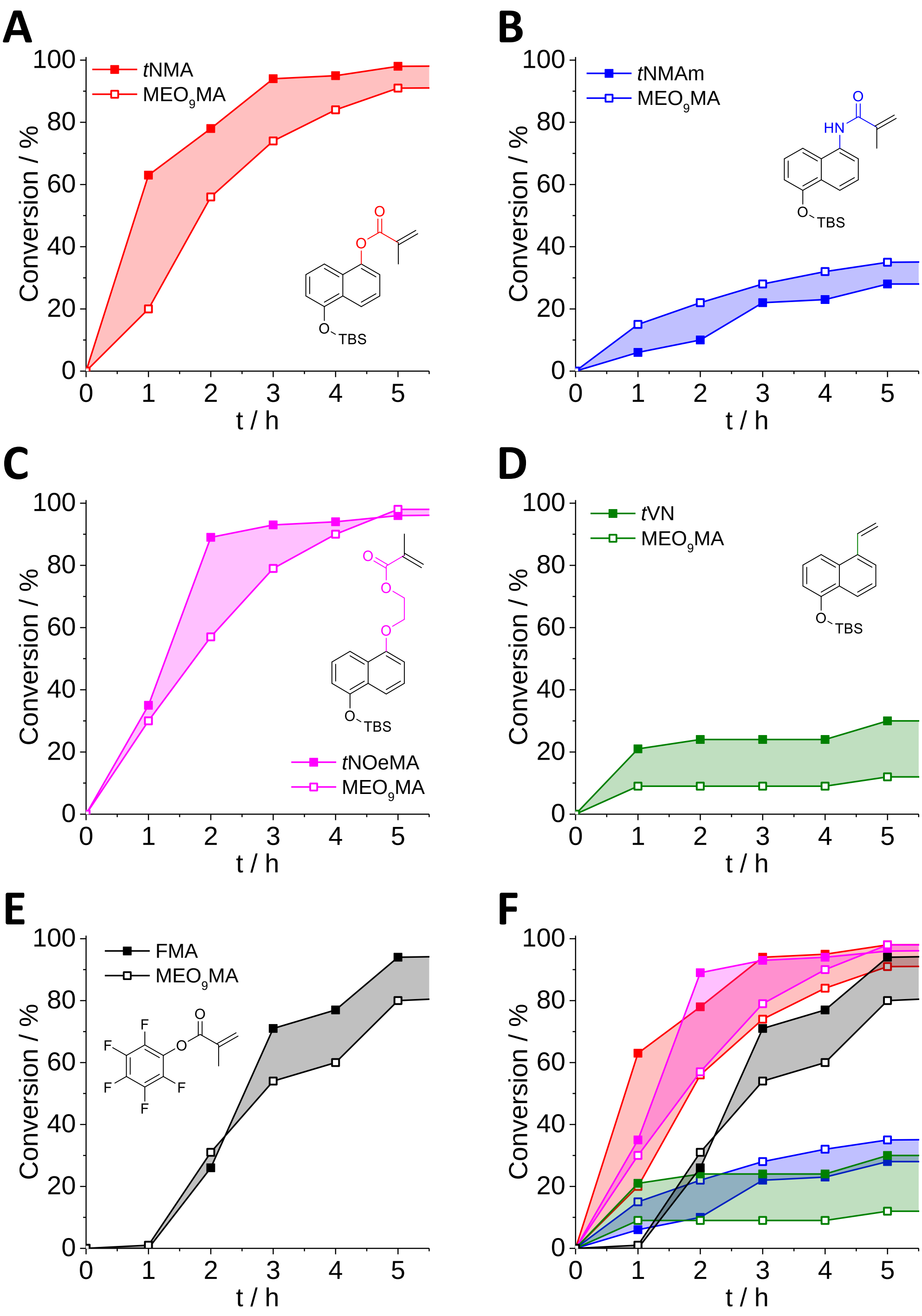

Figure S3: Conversion *vs* time plot for all five functional monomers (*t*NMA, *t*NMAm, *t*NOeMA, *t*VN, FMA) copolymerized with MEO$_9$MA via RAFT polymerization.

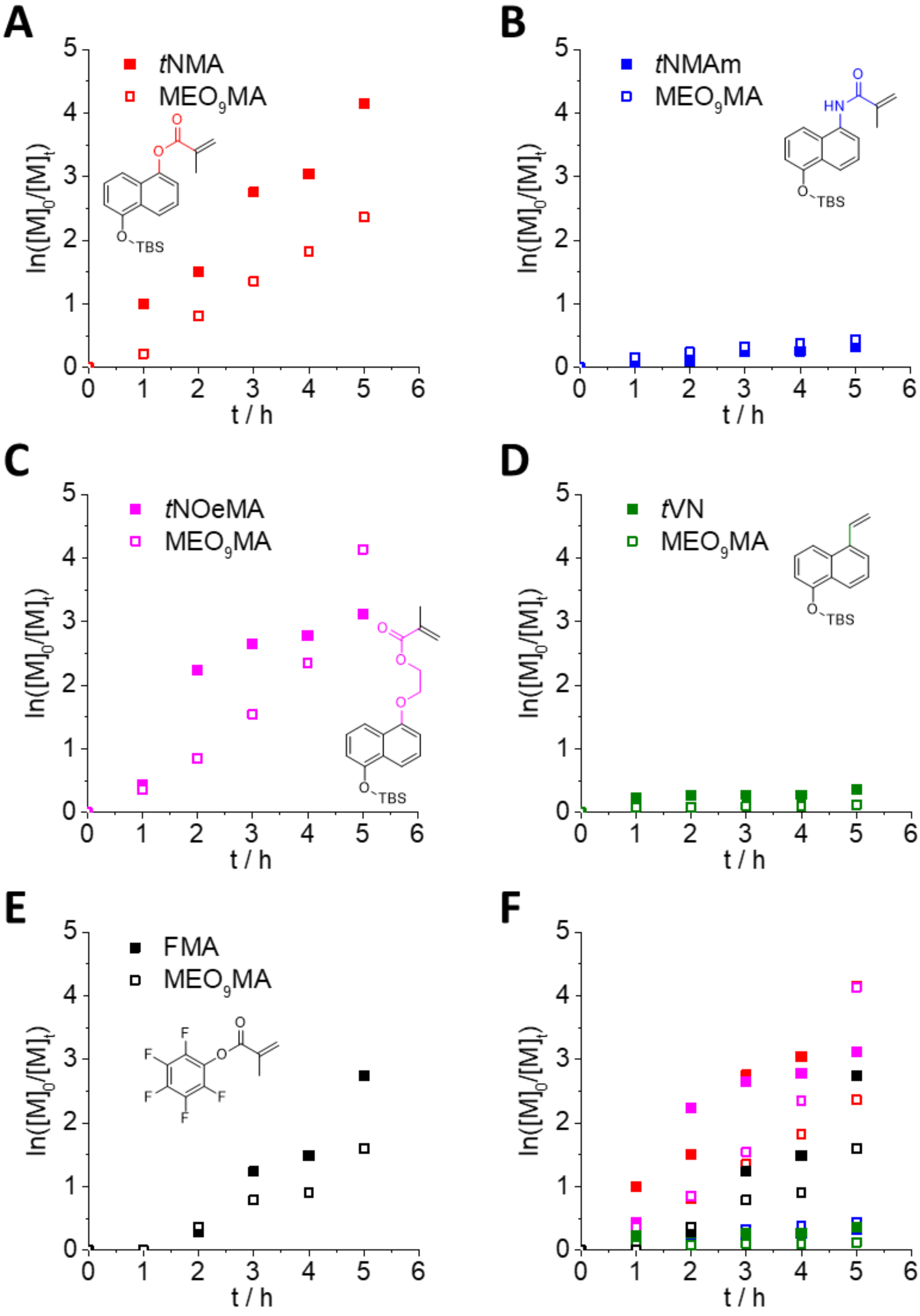

Figure S4: Semilogarithmic plot for all five comonomers (*t*NMA, *t*NMAm, *t*NOeMA, *t*VN, FMA) in a copolymerization with MEO_9MA.

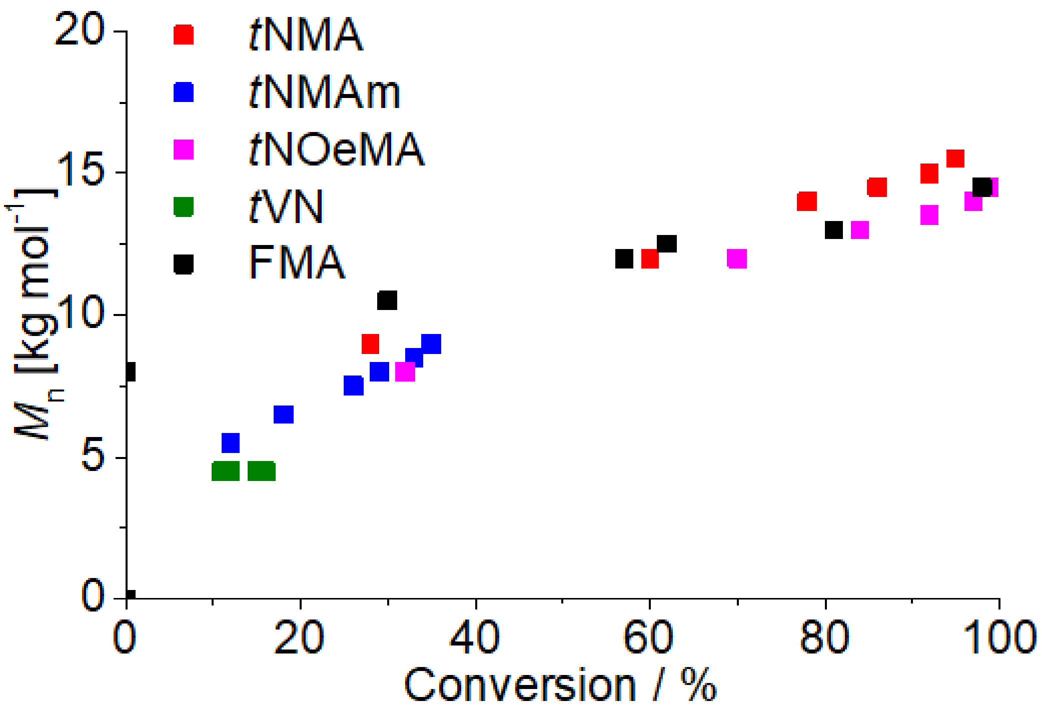

Figure S5: ***M_n vs.*** **conversion plot for all five comonomers (*t*NMA, *t*NMAm, *t*NOeMA, *t*VN, FMA) in a copolymerization with MEO_9MA.**

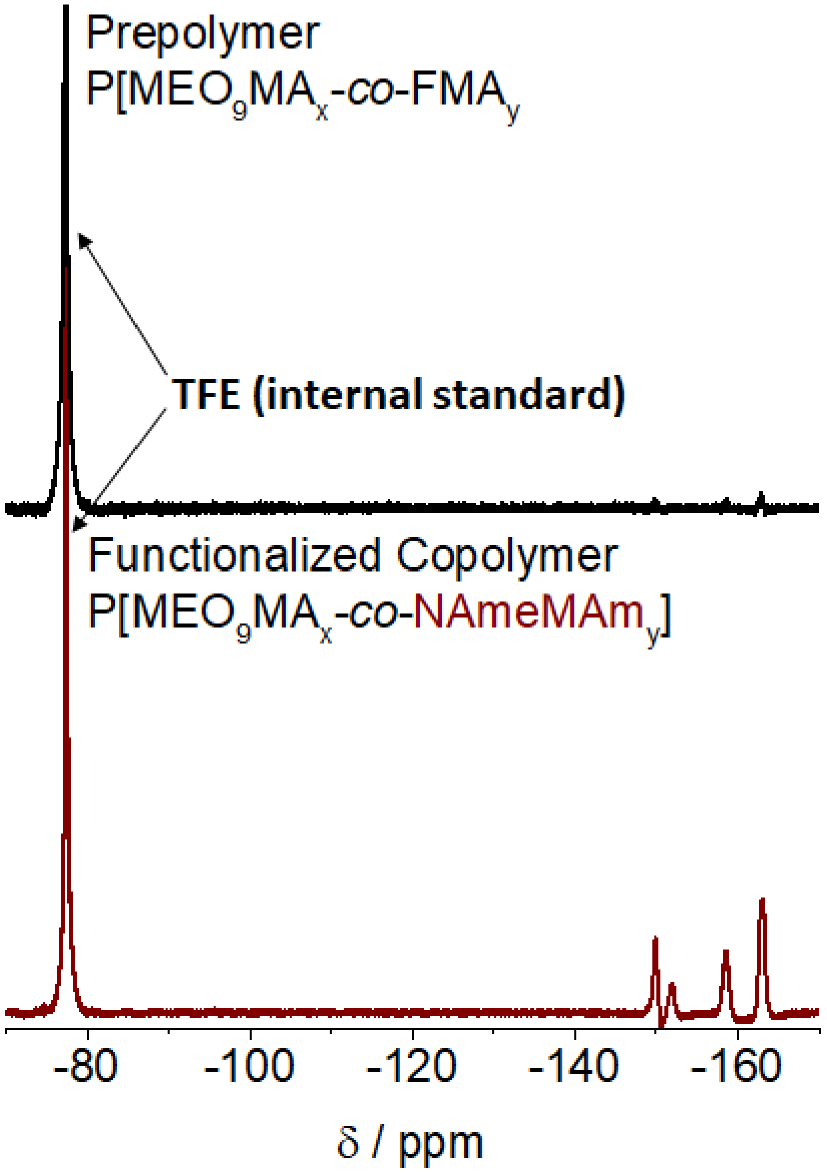

Figure S6: ^{19}F-NMR spectra of P[MEO$_9$MA$_x$-*co*-FMA$_y$/NAmeMAm$_y$] before (solid black line) and after post-polymerization modification (solid brown line) in CD_2Cl_2 using TFE as an internal standard.

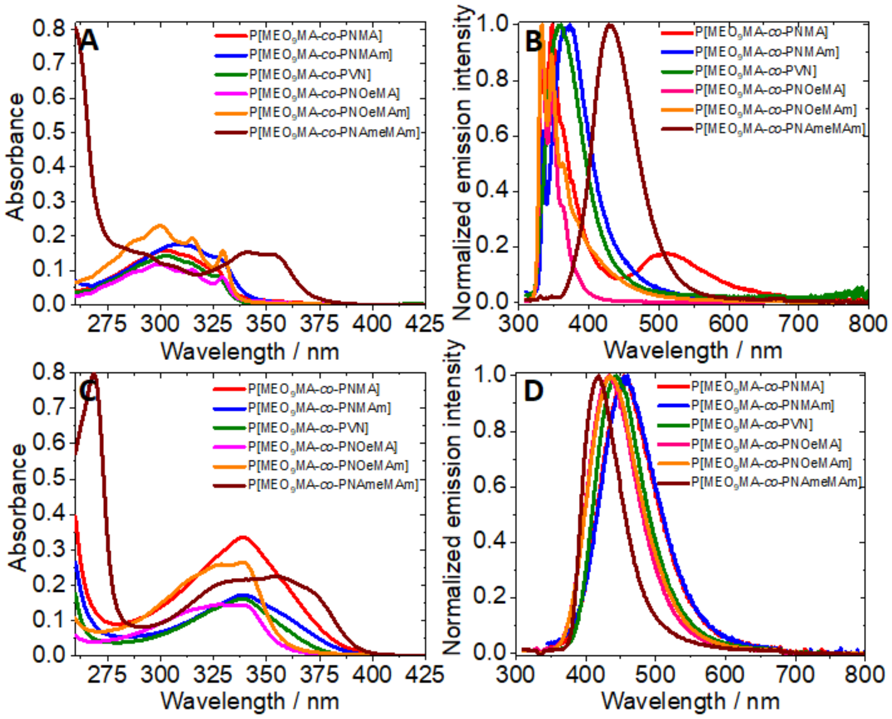

Figure S7: Compilation of UV/vis absorption and emission spectra of all investigated copolymers in acidified H_2O (A+B) and basic solutions (C+D).

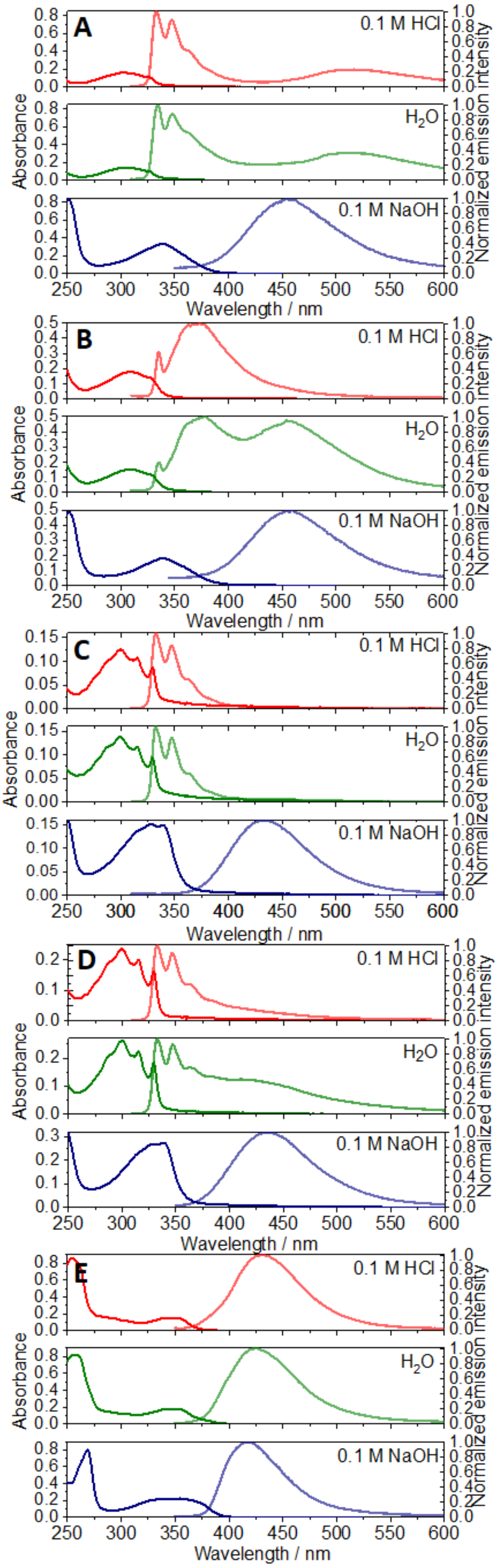

Figure S8: UV/vis absorption and emission (light colored) spectra of (A) P[MEO$_9$MA$_x$-*co*-NMA$_y$], (B) P[MEO$_9$MA$_x$-*co*-NMAm$_y$], (C) P[MEO$_9$MA$_x$-*co*-NOeMA$_y$], (D) P[MEO$_9$MA$_x$-*co*-NOeMAm$_y$], and (E) P[MEO$_9$MA$_x$-*co*-NAmeMAm$_y$] in aqueous media: 0.1 M HCl (red), H_2O (green) and 0.1 M NaOH (blue).

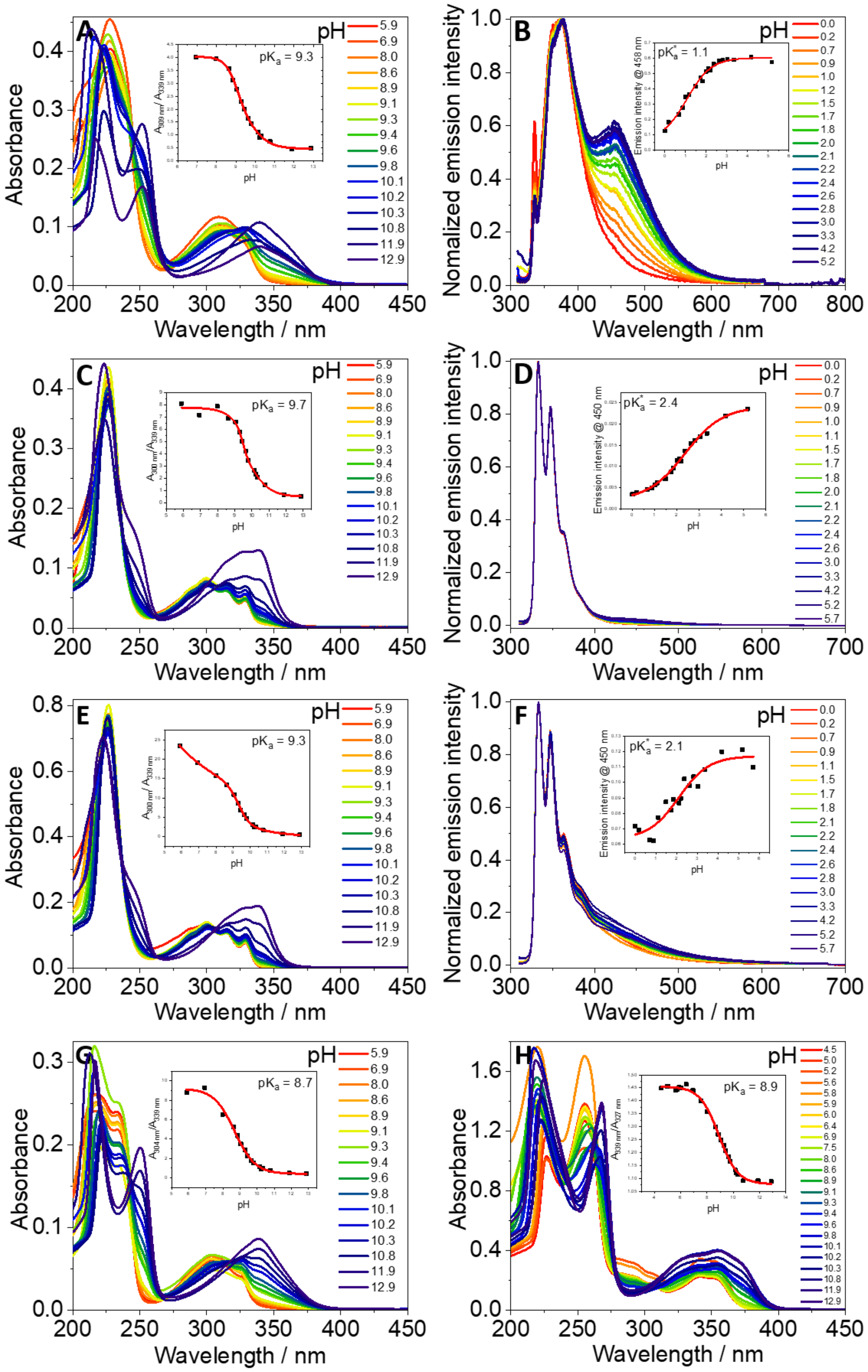

Figure S9: pH-dependent UV/vis absorption spectra and pH-dependent normalized emission spectra of (A+B) P[MEO$_9$MA$_x$-*co*-NMAm$_y$] (C+D) P[MEO$_9$MA$_x$-*co*-NOeMA$_y$], (E+F) P[MEO$_9$MA$_x$-*co*-NOeMAm$_y$] and the pH-dependent UV/vis absorption spectra of (G) P[MEO$_9$MA$_x$-*co*-NMA$_y$], and (H) P[MEO$_9$MA$_x$-*co*-NAmeMAm$_y$] in aqueous solutions. The respective insets show the estimated pK_a and pK_a^* values.

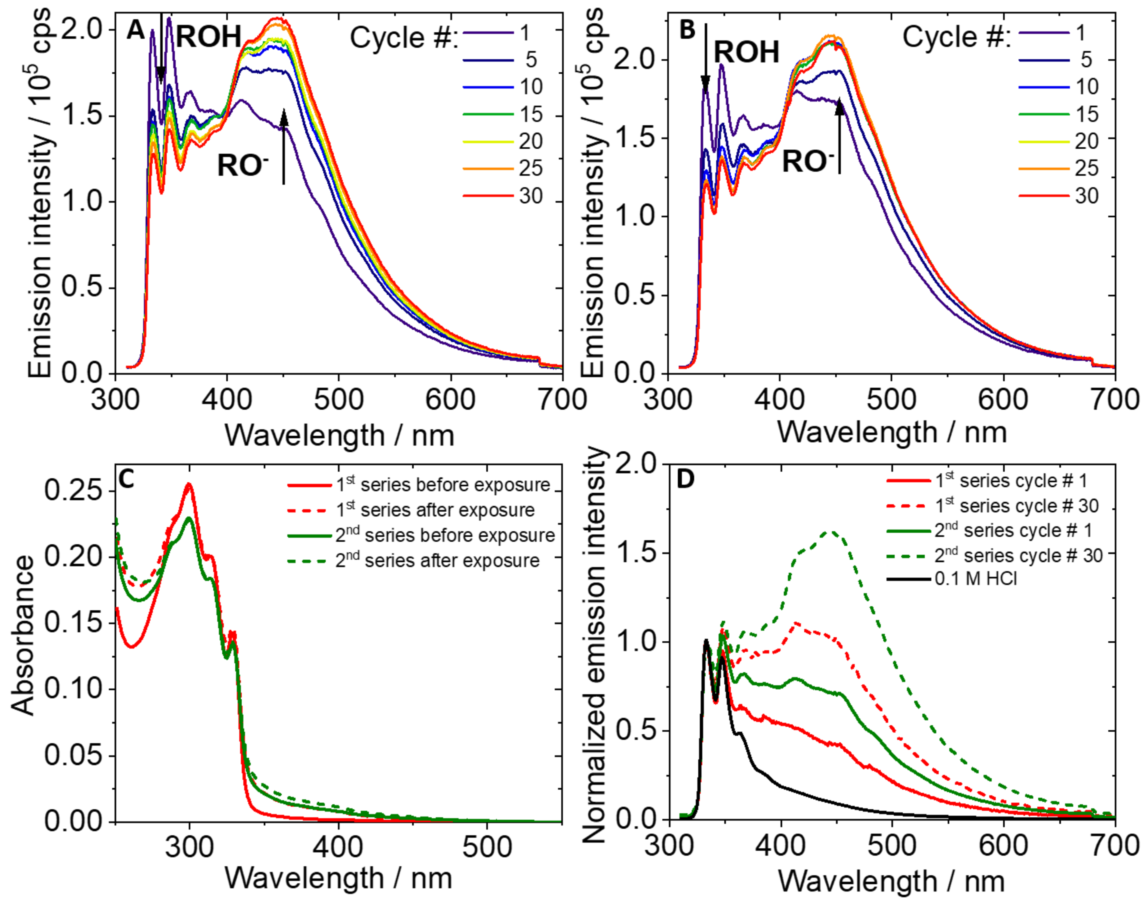

Figure S10: Emission spectra series of P[MEO$_9$MA$_x$-*co*-NOeMAm$_y$] measured under deaerated conditions in H_2O. The sample was irradiated continuously at λ_{exc} = 300 nm (P = 0.69 mW) with an exposure time per cycle of t = 4.5 min. (A) 1st emission series obtained from a freshly prepared sample. (B) 2nd emission series of the same sample solution stored in the dark between both measurements. (C) Emission spectra of the 1st and 30th cycle of the emission spectra series normalized to the maximum emission of pure ROH (λ_{max}^{em} = 334 nm). (D) UV/vis absorption spectra of P[MEO$_9$MA$_x$-*co*-NOeMAm$_y$] collected before and after illumination at λ_{exc} = 300 nm.

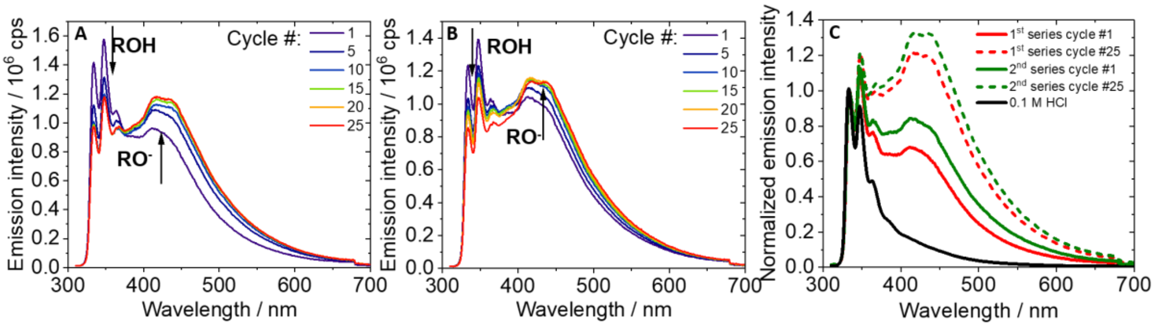

Figure S11: Series of emission spectra of P[MEO$_9$MA$_x$-*co*-NOeMAm$_y$] measured under deaerated conditions in H_2O. Sample was irradiated continuously at λ_{exc} = 300 nm (P = 0.21 mW) with an exposure time per cycle of t = 4.5 min. (A) 1st emission series obtained from a freshly prepared sample. (B) 2nd emission series of the same sample solution stored in the dark between both measurements. (C) Emission spectra of the illumination 1st and 25th cycle normalized on the maximum emission of pure ROH (λ_{max} = 334 nm).

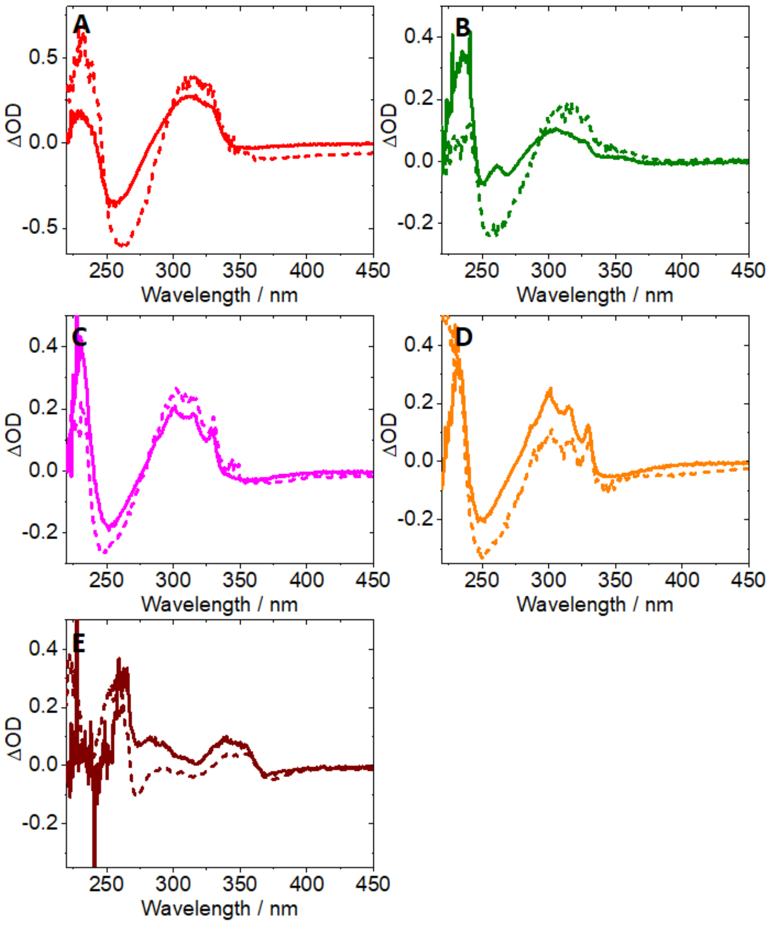

Figure S 12: UV/vis difference spectra of P[MEO$_9$MA$_x$-*co*-NMAm$_y$] (A), P[MEO$_9$MA$_x$-*co*-VN$_y$] (B), P[MEO$_9$MA$_x$-*co*-NOeMA$_y$] (C), P[MEO$_9$MA$_x$-*co*-NOeMAm$_y$] (D) and P[MEO$_9$MA$_x$-*co*-NAmeMAm$_y$] (E) obtained in UV/vis SCE measurement (solid lines) and UV/vis irradiation studies (dashed lines).

SYNTHESIS OF PHOTOACID-CONTAINING DIBLOCK QUARTERPOLYMERS

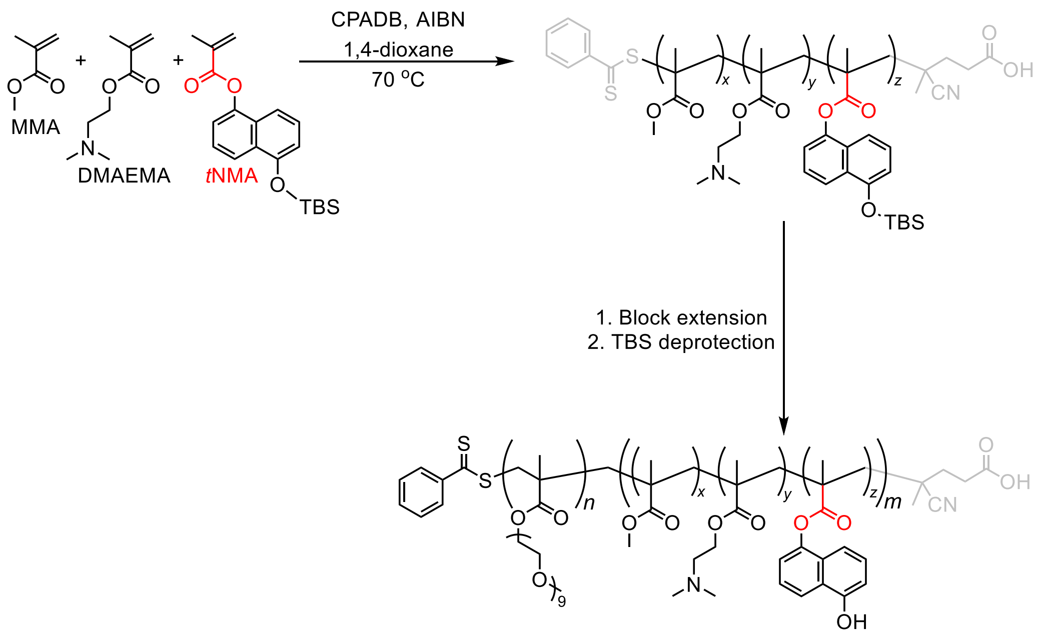

Figure S13: Schematic showing the RAFT polymerization (AIBN, CPADB, 1,4-dioxane, 70°C) of the respective protected photoacid monomers *t*NMA copolymerized with MMA and DMAEMA and synthetic route used to access P[$M_{0.32}$-$D_{0.17}$-*t*$NMA_{0.19}$]-*b*-P[$MEO_9MA_{0.32}$] diblock quarterpolymer *via* RAFT polymerization (AIBN, 1,4-dioxane, 70 °C) and subsequent deprotection step (TBAF/acetic acid, THF, 0 °C).

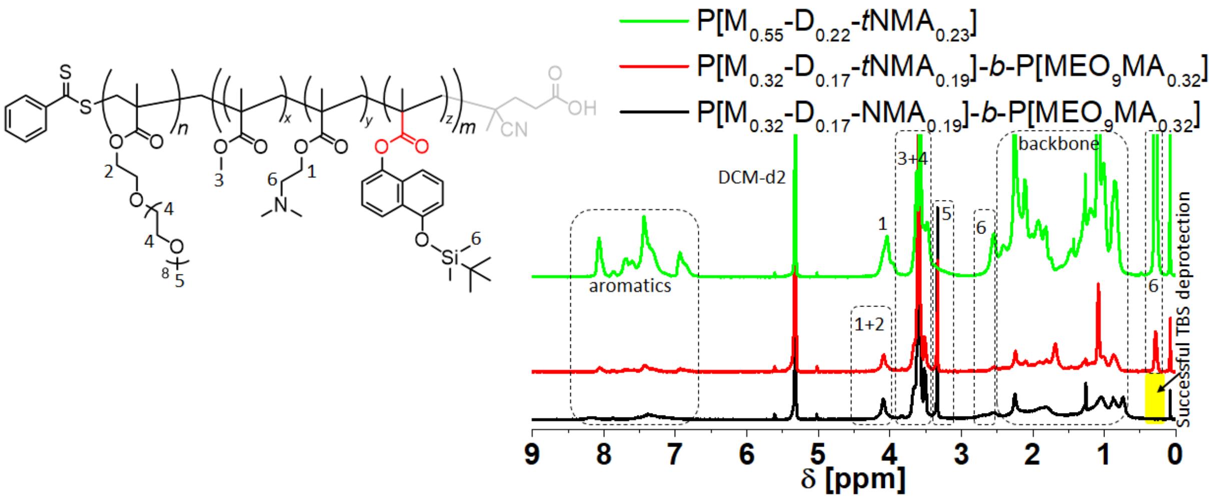

Figure S14: ^{1}H-NMR spectra of P[$M_{0.55}$-$D_{0.22}$-*t*$NMA_{0.23}$] (solid green line), P[$M_{0.32}$-$D_{0.17}$-*t*$NMA_{0.19}$]-*b*-P[$MEO_9MA_{0.32}$] (solid red line), P[$M_{0.32}$-$D_{0.17}$-$NMA_{0.19}$]-*b*-P[$MEO_9MA_{0.32}$] (solid black line) in CD_2Cl_2 with signal assignment and showing the successful deprotection of the TBS-protected block copolymer.

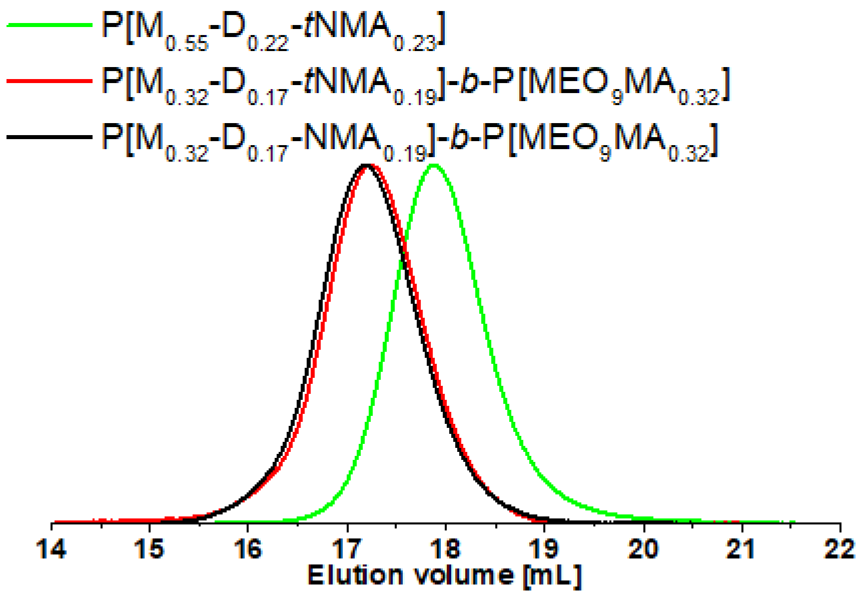

Figure S15: SEC (DMAc/LiCl) (PMMA calibration) elution traces for P[M$_{0.55}$-D$_{0.22}$-*t*NMA$_{0.23}$] (solid green line), P[M$_{0.32}$-D$_{0.17}$-*t*NMA$_{0.19}$]-*b*-P[MEO$_9$MA$_{0.32}$] (solid red line), P[M$_{0.32}$-D$_{0.17}$-NMA$_{0.19}$]-*b*-P[MEO$_9$MA$_{0.32}$] (solid black line).

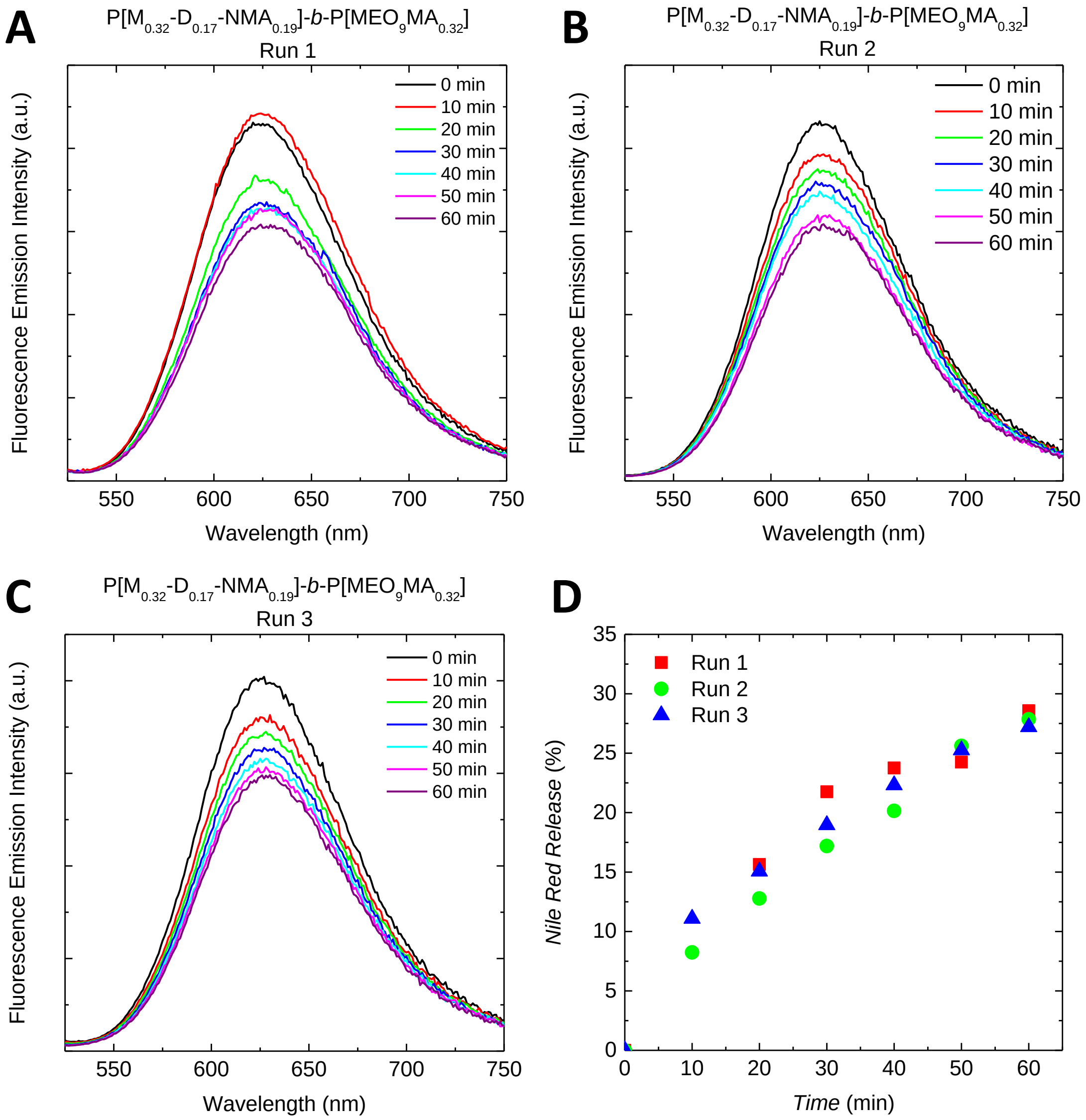

Figure S16: (A) Fluorescence emission spectra of NR-loaded micelles of P[M$_{0.32}$-D$_{0.17}$-NMA$_{0.19}$]-*b*-P[MEO$_{9}$MA$_{0.32}$] upon UV light irradiation showing the Run 1; (B) Run 2; (C) Run 3; NR release from P[M$_{0.32}$-D$_{0.17}$-NMA$_{0.19}$]-*b*-P[MEO$_{9}$MA$_{0.32}$] including all three runs.

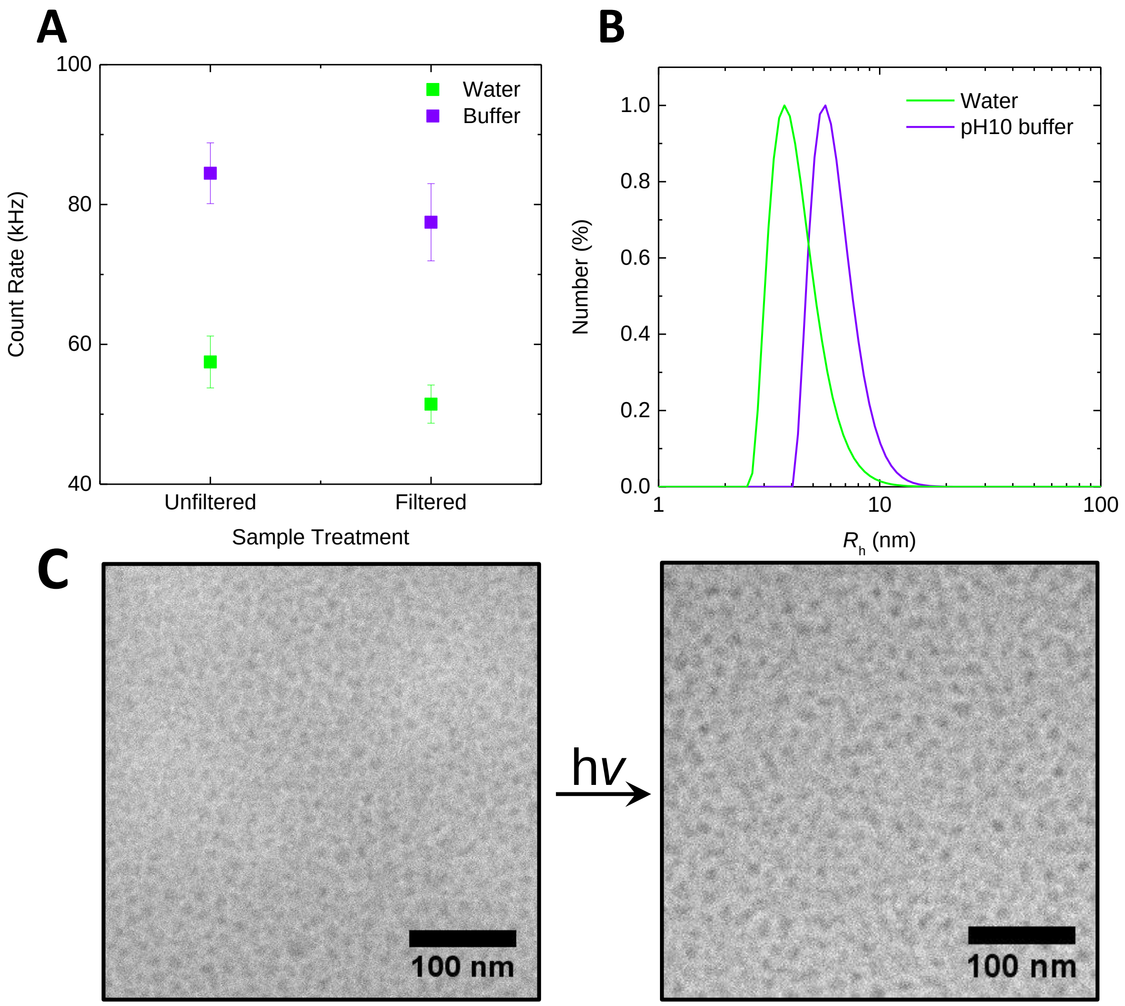

Figure S17 (A) Count rate and (B) number-weighted size distributions of P[$M_{0.32}$-$D_{0.17}$-$NMA_{0.19}$]-*b*-P[$MEO_9MA_{0.32}$] micelles in water and in a pH 10 buffer solution at a concentration of 1.0 mg mL^{-1}, in comparison to the (C) cryo-TEM images of the prepared micelles before and after photoactivation.
